# The effect of lifestyle interventions in women with polyendocrine metabolic ovarian syndrome: A systematic review and meta-analysis

**DOI:** 10.1371/journal.pmed.1004887

**Published:** 2026-09-23

**Authors:** Oladipupo Olalere, Min-Dee Koh, Katie Crabb, Olanike Ajijola, Saba Tariq, Chau Thien Tay, Aya Mousa, Soha Sobhy, Lynne Robinson, Rima Dhillon-Smith, Kathy Hoeger, Lisa Moran, Helena Teede, Shakila Thangaratinam

**Affiliations:** 1 Department of Metabolism and Systems Science, College of Medicine and Health, University of Birmingham, Birmingham, United Kingdom; 2 College of Medicine and Health, University of Birmingham, Birmingham, United Kingdom; 3 Department of Pharmacology, University Medical and Dental College, The University of Faisalabad, Faisalabad, Pakistan; 4 Monash Centre for Health Research and Implementation, Faculty of Clinical and Molecular Medicine, Monash University, Clayton, Victoria, Australia; 5 Diabetes Unit, Monash Health, Clayton, Victoria, Australia; 6 Birmingham Women’s Fertility Centre, Birmingham Women’s and Children’s NHS Foundation Trust, Birmingham United Kingdom; 7 Department of OBGYN, Division of Reproductive Endocrinology and Infertility, University of Rochester, Rochester, New York, United States of America; 8 Institute of Life Course and Medical Sciences, University of Liverpool, Liverpool, United Kingdom; 9 Liverpool Women’s NHS Foundation Trust, Liverpool, United Kingdom; 10 NIHR Applied Research Collaboration Northwest Coast, Liverpool, United Kingdom; University Hospital Ghent: Universitair Ziekenhuis Gent, BELGIUM

## Abstract

**Background:**

Lifestyle interventions are recommended as first-line treatment in polyendocrine metabolic ovarian syndrome (PMOS), but their effectiveness across various outcomes remains unclear. In this systematic review, we evaluate the effectiveness of lifestyle interventions on reproductive, anthropometric, and metabolic outcomes.

**Methods and findings:**

We searched Medline, EMBASE, All EBM, PsycInfo, and CINAHL to June 2026 for studies examining lifestyle intervention compared with minimal or no treatment (usual care) in PMOS. Meta-analyses were conducted using random-effects model to estimate pooled odd ratios (OR) with 95% confidence intervals (CI). We conducted subgroup analyses to evaluate differences by intervention type and duration, and by baseline body mass index (BMI).

A total of 34 studies (1,871 participants) were included from the retrieved 1,798 publications. Women who participated in lifestyle interventions were more likely to achieve regular menstrual cycles OR 4.03 (95% CI [1.16, 14.05] *I*^2^ = 73%, 5 studies, 237 women), and have reduced waist circumference mean difference (MD) −2.38 (95% CI [−4.68, −0.08] *I*^2^ = 90%, 18 studies, 915 women), fasting glucose MD −0.13 (95% CI [−0.25, −0.02] *I*^2^ = 86%, 22 studies, 1,045 women) and fasting insulin MD −6.08 (95% CI [−9.86, −2.30] *I*^2^ = 71%, 18 studies, 824 women) compared to women who had minimal or no treatment. Subgroup analyses showed that diet interventions had greater effect on reducing fasting glucose levels while exercise was most effective at reducing waist circumference and fasting insulin. In women with PMOS who were overweight or obese, lifestyle interventions led to greater reduction in weight, waist/hip ratio, fasting glucose, fasting insulin, total testosterone, total cholesterol, and low-density lipoprotein. The main limitation of this review is the high between-study heterogeneity in pooled estimates across many of the outcomes.

**Conclusion:**

Our review found that lifestyle interventions were associated with improved menstrual regularity and metabolic outcomes in women with PMOS, suggesting complementary benefits as no single intervention showed overall superiority. These findings support international recommendations, which endorse lifestyle interventions as first-line treatment, tailored to individual needs.

## Author summary

**Why was this study done?:**

- Polyendocrine metabolic ovarian syndrome (PMOS) is a common hormonal condition affecting up to around 10%–13% of women of reproductive age and is linked with irregular menstrual cycle, metabolic problems, and long-term cardiometabolic risk.
- Lifestyle interventions (diet, physical activity, behaviour change) are recommended as first-line treatment in PMOS, but the overall effectiveness and the best type and duration of these interventions to achieve desired reproductive and metabolic outcomes remain uncertain.
- An updated comprehensive synthesis of evidence from randomised trials was needed to inform clinical guidelines and to clarify which group of women benefit most from specific lifestyle interventions.

**What did the researchers do and find?:**

- We systematically reviewed and meta-analysed randomised controlled trials, comparing lifestyle interventions with minimal or no interventions. This review provides comprehensive subgroup analyses by intervention type, duration and baseline BMI.
- We found that overall, lifestyle interventions were associated with increased odds of achieving regular menstrual cycles, and with reductions in central adiposity (waist circumference), fasting glucose, and fasting insulin compared with minimal treatment.
- Exercise-based intervention, but not diet or behaviour-only interventions, improved body mass index, waist circumference, and fasting insulin, whereas dietary and combined interventions were particularly effective in lowering fasting glucose.
- In women with overweight or obesity, lifestyle interventions additionally improved weight, waist–hip ratio, total testosterone, and lipid profile, suggesting cardiometabolic benefit in this subgroup.

**What do these findings mean?:**

- These findings support lifestyle intervention as an essential first-line component of PMOS care, reinforcing current international guideline recommendations to prioritise individually tailored diet and exercise support in symptomatic women.
- Different lifestyle strategies may be more appropriate for different goals, with exercise particularly relevant for improving adiposity and insulin resistance, and dietary change more important for glycaemic control, especially in women with overweight or obesity.
- Given that many included trials had methodological limitations and heterogeneity in intervention content and delivery, more rigorous, longer-term studies are needed to define the most effective and sustainable lifestyle strategies, including their impact on pregnancy and birth outcomes.

## Introduction

Polyendocrine metabolic ovarian syndrome (PMOS) is a common, heterogeneous endocrine-metabolic disorder affecting 10% − 13% of women of reproductive age [1,2]. This important, yet often under recognised public health problem, confers a range of reproductive, cardiometabolic, dermatologic and psychological sequelae [1]. PMOS accounts for approximately 40% of women presenting with subfertility [3], and over 90% of women with anovulation attending infertility clinics [4]. Moreover, chronic oligo-anovulation results in prolonged exposure to unopposed oestrogen, thus increasing the risk of endometrial hyperplasia and cancer. Pregnancy in women with PMOS is independently associated with increased maternal and perinatal risks, which are further exacerbated by higher body mass index (BMI) and gestational weight gain. Consequently, PMOS is considered a high-risk pregnancy condition, where early and sustained lifestyle modifications may offer potential benefits across reproductive health [5]. An international, multidisciplinary consensus recently adopted the term polyendocrine metabolic ovarian syndrome (PMOS) to better reflect the complex endocrine, metabolic, and reproductive features of the condition. This terminology replaces polycystic ovary syndrome (PCOS) and aims to reduce the stigma and inaccuracies associated with the previous name [6].

While the exact cause of PMOS remains unclear, it is thought to involve a combination of genetic, environmental and lifestyle factors [7]. Hallmark features of PMOS include hyperinsulinemia, hyperandrogenism and neuroendocrine hormone abnormalities, which contribute to downstream metabolic and reproductive complications. This complex hormonal milieu contributes to a high prevalence of cardiometabolic disorders in PMOS, including obesity, dyslipidaemia, hypertension, diabetes, gestational diabetes and cardiovascular disease [8–10]. Underpinning many of these metabolic and endocrine disturbances is insulin resistance, an intrinsic pathological factor which is exacerbated by obesity-related insulin resistance in PMOS [11,12]. Given its central role, interventions targeting insulin resistance are key to improving both clinical and metabolic outcomes in most women with PMOS [11,13].

Lifestyle interventions, including exercise and diet modification, are recommended as first-line management for symptomatic women with PMOS [1]. These strategies are commonly used for both weight management (including weight loss, weight maintenance or prevention of excessive weight gain) and overall general cardiometabolic health improvement [14]. Recent reviews [1,15,16] have reported beneficial effects of lifestyle interventions on reproductive, metabolic, and anthropometric outcomes in women with PMOS. However, existing evidence is limited by the number of studies, variation in intervention types, high risk of bias, and lack of granularity regarding optimal types and durations of interventions [17].

Given the rapidly evolving nature of research in this field, this review aims to provide more comprehensive evaluation of the effectiveness of lifestyle interventions on reproductive, anthropometric, and metabolic outcomes in women with PMOS, and to explore whether these effects vary by intervention type, duration, or baseline body mass index (BMI).

## Methods

We conducted and reported this systematic review and meta-analysis in accordance with the Preferred Reporting Items for Systematic Reviews and Meta-Analyses (PRISMA) guidelines [18] (S1 Checklist). We prospectively registered the study protocol (PROSPERO CRD42024565919).

### Search strategy and study selection

We updated our previous search for a 2022 technical report for the International Evidence-based Guideline for the Assessment and Management of PCOS [17] by searching Ovid Medline, EMBASE, All EBM, PsycInfo, and CINAHL from 1 January 2022 to 9 June 2026, using the same search terms (S1 Appendix). There were no material differences in the eligibility criteria, screening processes, or data synthesis methods between this review and the 2022 technical report. Two reviewers (OO and STq) independently identified eligible studies based on the predefined criteria by screening the titles and abstracts and then assessing the full text. We resolved disagreements through discussion and consensus.

We included all randomised controlled trials (RCTs) investigating the effects of lifestyle interventions on fertility, hormonal, anthropometric and/or metabolic outcomes in women with PMOS and applied no language restrictions. The diagnosis of PMOS across the included studies was according to NIH 1990, Rotterdam 2003 [19], or AES-PCOS 2006 criteria [20]. Eligible lifestyle interventions included exercise, diet, behavioural management or a combination of these measures for a minimum duration of two weeks. We defined the comparator as minimal treatment which comprises no treatment/usual care or standard unstructured minimal dietary, exercise or behavioural advice. Following the CORE outcomes in PCOS [21], we planned to assess a broad range of reproductive outcomes (live birth, clinical pregnancy, miscarriage, biochemical pregnancy, ovulation, ovarian hyperstimulation, oocyte yield, oocyte maturation rate, duration of ovarian stimulation, fertilisation rate, ovarian hyperstimulation syndrome, preterm birth, gestational age at birth, gestational diabetes mellitus, pre‑eclampsia, intra‑uterine growth restriction, fetal macrosomia, stillbirth, neonatal death, perinatal death, NICU admission, cesarean section and shoulder dystocia). However, in the included trials, extractable reproductive data were almost entirely limited to regular and irregular menstrual cycles and, in one study, conception resulting in live birth. We therefore treated menstrual cycle regularity as the primary reproductive outcome and conception/live birth as exploratory outcome. Other non-reproductive outcomes include weight, BMI, waist circumference, waist-hip ratio, sex hormone-binding globulin, total testosterone, free androgen index, fasting glucose, fasting insulin, and lipid profile (total cholesterol, triglycerides, high-density and low-density lipoprotein cholesterol).

### Quality assessment and data extraction

We used the Cochrane Risk of Bias (ROB) 2.0 tool [22] to evaluate study-level risk of bias across five domains: randomisation process, deviations from intended intervention, incomplete outcome data, outcome measurement, and selective reporting. Two reviewers independently assessed study quality, and any disagreements were resolved through consultation with a third, independent reviewer. We considered a study as having a low risk of bias if the trial was awarded low risk of bias in all domains, trials with some concerns of bias in one domain were considered as having a moderate risk of bias, and trials with a high risk of bias in at least one domain or some concerns of bias in multiple domains were considered as having a high risk of bias.

Two reviewers independently extracted data using a pre-designed data extraction sheet. Relevant study characteristics were extracted including year of publication, country, participant inclusion and exclusion criteria, study intervention, number of recruited participants, and details of comparator group(s). Dichotomous data were extracted as 2 × 2 tables, while means and standard deviations (SD) were extracted for continuous data. Where medians and interquartile ranges were reported instead of means and SD, we used medians in place of means and calculated the SD using the formula of SD = (third quartile-first quartile)/1.35. Disagreements were resolved by consensus.

We used Grading of Recommendations Assessment, Development and Evaluation (GRADE) to rate the certainty (high, moderate, low, very low) of evidence for all outcomes.

### Statistical analysis

To assess differences between the intervention and control (minimal treatment) groups, we calculated effect sizes for each dichotomous and continuous outcome, reported as odds ratios (OR) or weighted mean differences (MD), respectively, with corresponding 95% confidence intervals (CI). The studies were weighted based on the inverse of the variance for the evaluated measure with a random-effects model. Heterogeneity was summarised using *I*^*2*^ values, defined as low, moderate, or high heterogeneity according to the cut-offs of 25%, 50%, and 75%, respectively. We determined statistical significance using a two-tailed threshold of *P* < 0.05. All analyses were conducted on Review Manager version 5 [23].

### Sensitivity and subgroup analyses

We performed sensitivity analyses whereby data from studies assessed to have high risk of bias were excluded to gage their impact on the findings. Blinding was not used in the sensitivity analysis as it was not possible to blind participants and the intervention provider due to the interactive nature of the interventions.

Where data were available, we conducted subgroup analyses to determine the specific evidence within the following subgroups.

- Component of intervention (dietary alone versus exercise alone versus behavioural intervention alone versus combined intervention).
- Duration of intervention (short: less than four weeks, medium: four weeks to three months, long: greater than three months).
- Studies where the inclusion criterion was overweight participants versus studies with no specific inclusion weight criterion.

We did not use any artificial intelligence tool or technology in the design, screening, data extraction, analysis, interpretation, or reporting of this study.

## Results

**Fig 1:**
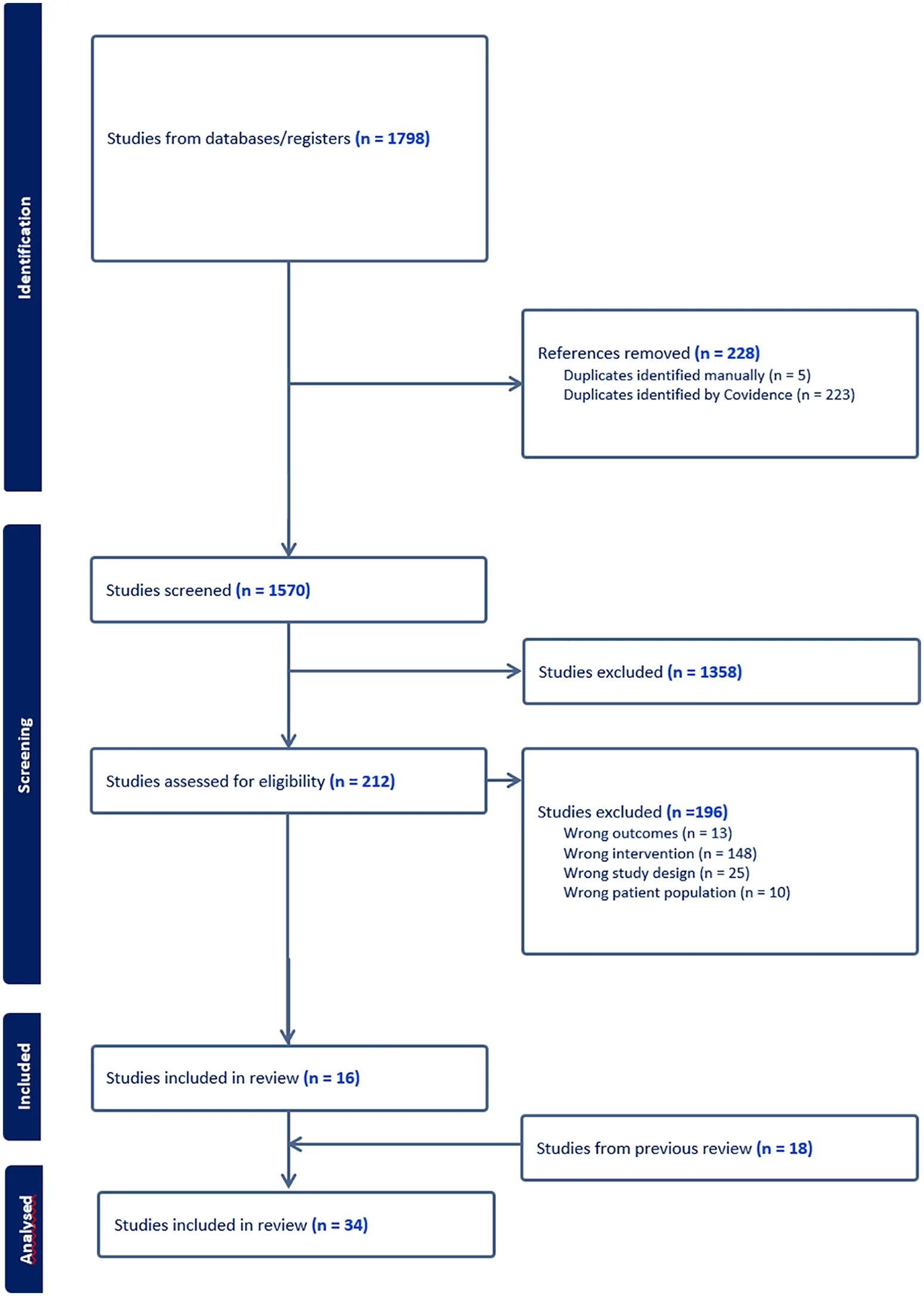
Preferred Reporting Items for Systematic Reviews and Meta-analyses (PRISMA) 2020 flow diagram.

Of the 1,798 records retrieved, 34 studies (16 retrieved in the current search and 18 from the 2022 technical report) were included with a total of 1871 participants (Fig 1).

### Study characteristics

Seventeen studies were from high income counties including Australia [24,25], China [26], Denmark [27], Greece [28], Italy [29–31], the Netherlands [32], Norway [33], Sweden [34,35], UK [36], and USA [37–40], eight were from two upper middle income countries Brazil [41–46] and Turkey [47,48], and the remaining nine studies were conducted in Iran [49–53] and India [54–57] (S2 Appendix).

**Fig 2:**
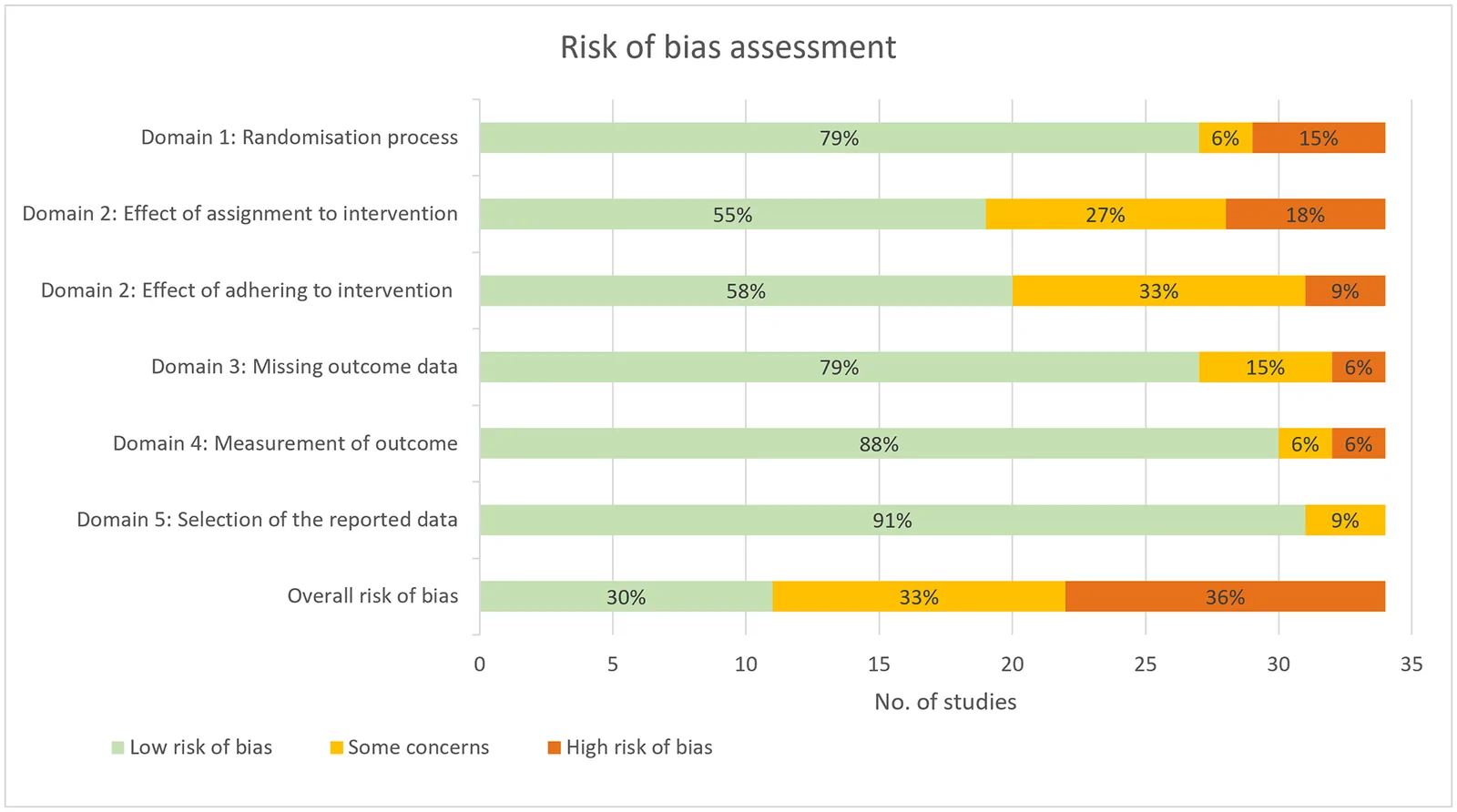
Risk of bias assessment of included studies.

Using the Cochrane ROB2 tool [22], 79% (27/34) of the included studies had a low risk of bias for the randomisation process, 56% (19/34) for effect of assignment to intervention, 59% (20/34) for effect of adhering to intervention, 79% (27/34) for missing outcome data, 88% (30/34) for meassurement of outcome, and 91% (31/34) for selection of reported data. Thirty-five percent (12/34) of the included studies had an overall high risk bias (Fig 2, S3 Appendix).

We assessed the quality of the outcomes using the GRADE ranking system (S4 Appendix).

### Effect of lifestyle interventions on reproductive outcomes

Women with PMOS who had lifestyle interventions had increased odds of achieving regular menstrual cycles OR 4.03 (95% CI [1.16, 14.05] *I*^*2*^ = 73%, 5 studies, 237 women) than women who had minimal treatment.

Only one of the included studies [58] reported on conception resulting in live birth. This follow-up study on pregnancy outcomes from a RCT assessing lifestyle interventions (a combination of normo-caloric diet, exercise according to the World Health Organization’s Global Recommendations, and cognitive behavioural therapy) in overweight/obese women with PMOS found that 39.8% (49/123) of those in the intervention group achieved pregnancy which resulted in live birth, compared to 38.3% (23/60) of women in the usual care group [58]. The finding was not statistically significant.

No other planned fertility or pregnancy outcomes had sufficient or consistent reporting to allow quantitative synthesis.

### Effect of lifestyle interventions on anthropometric and metabolic outcomes

Compared to women with PMOS who had minimal treatment, those who had lifestyle interventions exhibited greater reductions in waist circumference MD −2.38 (95% CI [−4.68, −0.08] *I*^2^ = 90%, 18 studies, 915 women), fasting glucose MD −0.13 (95% CI [−0.25, −0.02] *I*^2^ = 86%, 22 studies, 1,045 women), and fasting insulin MD −6.08 (95% CI [−9.86, −2.30] *I*^2^ = 71%, 18 studies, 824 women). There were no significant differences between groups when comparing effects on weight, BMI, waist circumference, waist-hip ratio, sex hormone binding globulin, total testosterone, free androgen index, or lipid profile (Table 1).

**Table 1:** Summary effects of lifestyle interventions on anthropometric and metabolic outcomes.

Outcome	Studies	Participants	Mean Difference (95% CI)	*I*^2^ (%)
Weight (kg)	21	1,065	−1.97 [−4.04, 0.11]	86
Body mass index (kg/m^2)^	28	1,319	−0.5 [−1.04, 0.05]	71
**Waist circumference (cm)**	**18**	**915**	**−2.38 [−4.68, −0.08]**	**90**
Waist-hip ratio	14	711	−0.02 [−0.04, 0.01]	86
Sex hormone-binding globulin (nmol/L)	14	716	2.67 [−0.00, 5.34]	30
Total testosterone (nmol/L)	19	982	−0.09 [−0.27, 0.09]	73
Free Androgen Index	12	560	−0.48 [−1.16, 0.21]	43
**Fasting glucose (mmol/L)**	**22**	**1,045**	**−0.13 [−0.25, −0.02]**	**86**
**Fasting insulin (pmol/L)**	**18**	**824**	**−6.08 [−9.86, −2.30]**	**71**
Total cholesterol (mmol/L)	17	747	−0.09 [−0.20, 0.03]	38
High-density lipoprotein cholesterol (mmol/L)	15	666	0.01 [−0.04, 0.05]	28
Low-density lipoprotein cholesterol (mmol/L)	15	641	−0.13 [−0.27, 0.01]	66
Triglycerides (mmol/L)	16	707	0.07 [−0.02, 0.16]	32

### Sensitivity analyses

Following the exclusion of studies with an overall high risk of bias rating, only the beneficial effect of lifestyle interventions on fasting insulin MD −5.71 pmol/L (95%CI [−9.60, −1.83] *P* = 0.004) compared with minimal treatment persisted, while differences in fasting glucose and waist circumference were attenuated MD −0.07 (95% CI [−0.20, 0.06]) and MD −1.94 (95% CI [−3.93, 0.05]), respectively (Table 2).

**Table 2:** Sensitivity analysis of the effect of lifestyle interventions on anthropometric and metabolic outcomes.

Outcome	Studies	Participants	Mean Difference (95% CI)	*I*^2^ (%)
Weight (kg)	16	804	−0.65 [−2.04, 0.75]	58
Body mass index (kg/m^2)^	20	917	−0.25 [−0.65, 0.16]	37
Waist circumference (cm)	12	618	−1.94 [−3.93, 0.05]	66
Waist-hip ratio	9	489	−0.02 [−0.05, 0.01]	87
Sex hormone-binding globulin (nmol/L)	11	608	2.43 [−0.65, 5.51]	34
Total testosterone (nmol/L)	13	665	0.07 [−0.16, 0.30]	75
Free Androgen Index	10	464	−0.49 [−1.23, 0.25]	50
Fasting glucose (mmol/L)	15	729	−0.07 [−0.20, 0.06]	73
**Fasting insulin (pmol/L)**	**14**	**670**	**−5.71 [−9.60, −1.83]**	**75**
Total cholesterol (mmol/L)	11	449	−0.01 [−0.17, 0.15]	43
High-density lipoprotein cholesterol (mmol/L)	10	390	0.01 [−0.06, 0.07]	32
Low-density lipoprotein cholesterol (mmol/L)	11	449	−0.07 [−0.23, 0.019]	38
Triglycerides (mmol/L)	11	449	0.08 [−0.03, 0.19]	28

However, the effect of lifestyle interventions on androgen markers and lipid profiles remained inconclusive.

### Subgroup analyses

#### Intervention type subgroups.

There was evidence of subgroup differences by types of intervention for BMI, waist circumference, fasting glucose, fasting insulin, and low-density lipoproteins (Table 3).

**Table 3:** Summary of effectiveness by types of interventions.

Outcome	Subgroup	Studies	Participants	Mean Difference (95% CI)	*I*^2^ (%)
Weight (kg)	Exercise	13	634	−2.33 [−5.42, 0.75]	83
	Diet	5	317	−0.82 [−3.49, 1.85]	46
	Behaviour changes	2	96	−0.74 [−2.43, 0.95]	2
	Combined	1	18	1.00 [−17.10, 19.10]	–
BMI	**Exercise**	**16**	**725**	**−0.81 [−1.60, −0.02]**	**56**
	Diet	7	431	0.18 [−1.42, 1.77]	86
	Behaviour changes	3	134	−0.44 [−1.68, 0.80]	52
	Combined	2	31	0.95 [−4.09, 5.98]	0
Waist circumference (cm)	**Exercise**	**11**	**496**	**−3.17 [−5.61, −0.73]**	**87**
	Diet	6	401	−1.27 [−7.21, 4.68]	92
	Behaviour changes	0	0	–	–
	Combined	1	18	4.60 [−12.03, 21.23]	–
Waist-hip ratio	Exercise	8	360	−0.02 [−0.04, −0.00]	10
	Diet	5	339	−0.01 [−0.05, 0.03]	94
	Behaviour changes	1	12	–	–
	Combined	0	0	–	–
Sex hormone-binding globulin (nmol/L)	Exercise	8	439	1.12 [−3.22, 5.47]	16
	Diet	2	166	4.05 [−1.73, 9.83]	41
	Behaviour changes	2	80	3.73 [−5.30, 12.76]	81
	Combined	2	31	9.03 [−1.22, 19.28]	0
Total testosterone (nmol/L)	Exercise	10	538	−0.15 [−0.31, −0.00]	32
	Diet	5	333	0.09 [−0.43, 0.61]	92
	Behaviour changes	2	80	−0.80 [−1.78, 0.18]	–
	Combined	2	31	−0.23 [−0.79, 0.33]	0
Free Androgen Index	Exercise	8	439	−0.50 [−1.14, 0.13]	31
	Diet	1	22	−1.75 [−4.47, 0.97]	–
	Behaviour changes	1	68	1.60 [−0.14, 3.34]	–
	Combined	2	31	−3.60 [−7.89, 0.70]	20
Fasting glucose (mmol/L)	Exercise	12	535	−0.01 [−0.18, 0.16]	86
	**Diet**	**5**	**299**	**−0.38 [−0.60, −0.17]**	**65**
	Behaviour changes	2	80	−0.22 [−0.45, 0.01]	0
	**Combined**	**3**	**131**	**−0.42 [−0.44, −0.40]**	**0**
Fasting insulin (pmol/L)	**Exercise**	**11**	**485**	**−1.86 [−2.96, −0.76]**	**0**
	Diet	3	228	−20.42 [−46.63, 5.78]	90
	Behaviour changes	2	80	−10.57 [−27.58, 6.43]	0
	Combined	2	32	−1.72 [−10.19, 6.75]	0
Total cholesterol (mmol/L)	Exercise	11	511	−0.11 [−0.22, 0.01]	28
	Diet	4	205	0.12 [−0.33, 0.57]	71
	Behaviour changes	0	0	–	–
	Combined	2	31	−0.41 [−1.08, 0.27]	3
High-density lipoprotein cholesterol (mmol/L)	Exercise	11	511	0.00 [−0.05, 0.05]	32
	Diet	2	124	−0.01 [−0.08, 0.06]	14
	Behaviour changes	0	0	–	–
	Combined	2	31	0.08 [−0.11, 0.28]	54
Low-density lipoprotein cholesterol (mmol/L)	Exercise	11	511	−0.12 [−0.27, 0.04]	67
	Diet	2	99	0.03 [−0.45, 0.52]	69
	Behaviour changes	0	0	–	–
	**Combined**	**2**	**31**	**−0.66 [−1.25, −0.08]**	**16**
Triglycerides (mmol/L)	Exercise	11	511	0.04 [−0.04, 0.13]	19
	Diet	3	165	0.24 [−0.08, 0.56]	65
	Behaviour changes	0	0	–	–
	Combined	2	31	−0.06 [−0.44, 0.31]	21

**Fig 3:**
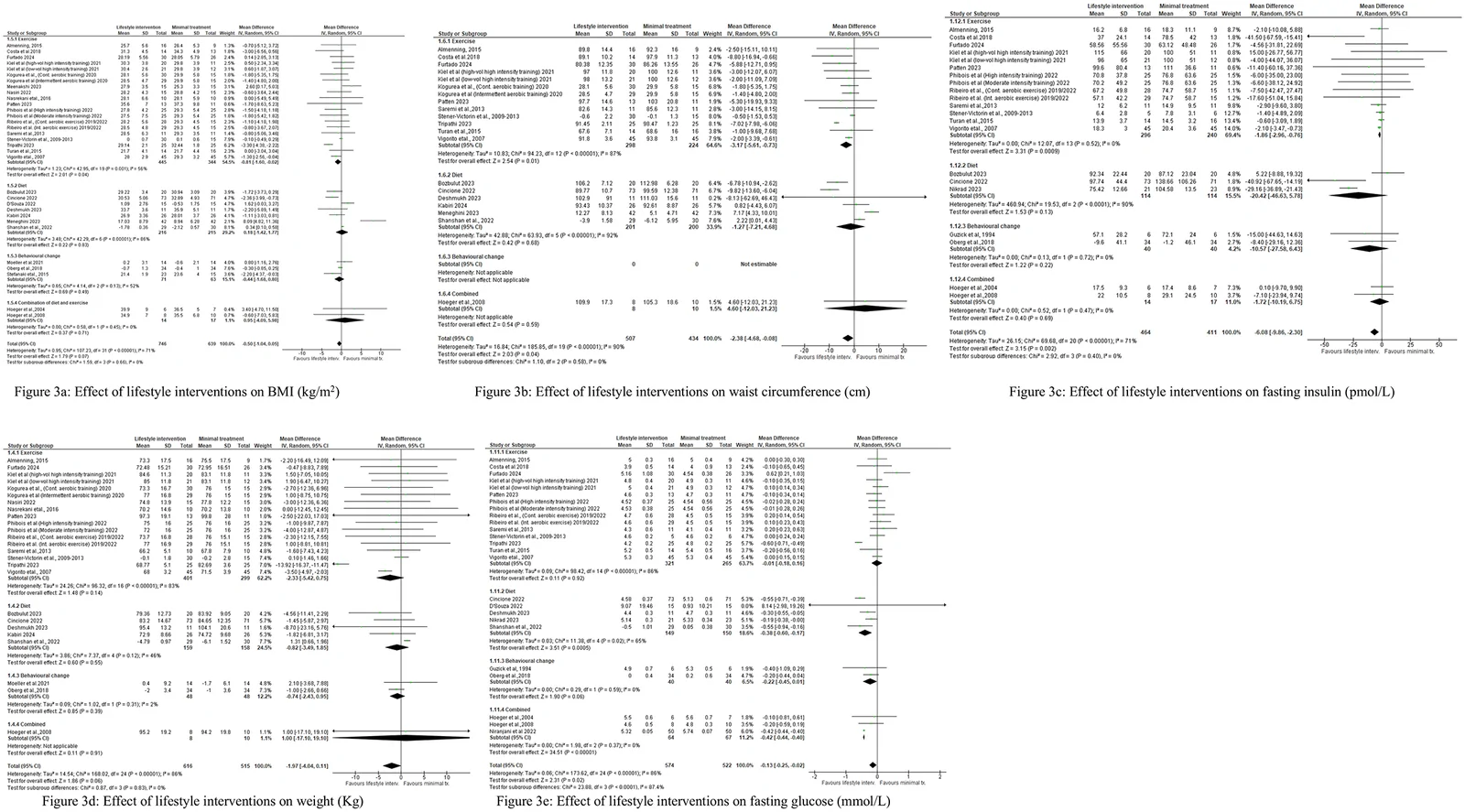
Effect of lifestyle interventions by intervention types.

Exercise-based interventions demonstrate greater reduction in post-intervention BMI MD −0.81 kg/m^2^ (95% CI [−1.60, −0.02] *P* = 0.04; Fig 3a), waist circumference MD −3.17 cm (95% CI –[5.61, −0.73] *P* = 0.01; Fig 3b), fasting insulin MD −1.86 pmol/L (95% CI [−2.96, −0.76] *P* = 0.0009; Fig 3c) compared with minimal treatment.

There were no significant differences in BMI in those who had diet modification MD 0.43 kg/m^2^ (95% CI [−1.42, 2.28] *P* = 0.65), behaviour change MD −0.44 kg/m^2^ (95% CI [−1.68, 0.08] *P* = 0.49), or a combined intervention MD 0.95 kg/m^2^ (95% CI [−4.09, 5.98] *P* = 0.71) versus those who had minimal treatment (Fig 3a). No differences in waist circumference were seen between women who had diet modification or combined interventions versus those who had minimal treatment, with a MD −1.77 cm (95% CI [−8.82, 5.28] *P* = 0.62) and MD 4.6 cm (95% CI [−12.03, 21.23] *P* = 0.59) (Fig 3b). Diet, behaviour change, or combined interventions did not show significant difference on fasting insulin when compared with minimal treatment (Fig 3c).

There were no significant differences in weight among women who had exercise, diet modification, behaviour change, or combined interventions versus those who had minimal treatment, with MD −2.33 kg, (95% CI [−5.42, 0.75] *P* = 0.14), −0.84 kg (95% CI [−4.10, 2.43] *P* = 0.61), −0.74 kg (95% CI [−2.43, 0.95] *p* = 0.39), and 1 kg (95% CI [−17.1, 19.1] *p* = 0.91), respectively (Fig 3d).

Diet modifications and combined interventions had significant reduction on fasting glucose levels compared to minimal treatment with MDs of −0.38 mmol/L (95% CI [−0.60, −0.17] *P* = 0.0005) and −0.42 mmol/L (95% CI [−0.44, −0.40] *P* < 0.00001), respectively. There were no differences in fasting glucose in women who had exercise or behaviour change interventions versus minimal treatment, with MDs of −0.01 mmol/L (95% CI [−0.18, 0.16] *P* = 0.92) and −0.22 mmol/L (95% CI [−0.45, 0.01] *P* = 0.06), respectively (Fig 3e).

Combined interventions demonstrate greater reduction in post-intervention low-density lipoproteins MD −0.66 mmol/L (95% CI [−1.25, −0.08] *p* = 0.03). However, no difference was seen in the level of low-density lipoprotein between women who exercise, diet or behaviour therapy alone compared to those who had minimal treatment (Table 3).

There were no statistically significant differences based on the type of lifestyle intervention for all other measured outcomes including waist-hip ratio, sex hormone-binding globulin, total testosterone, free androgen index, total cholesterol, high-density lipoprotein, and triglycerides (Table 3, S5 Appendix).

#### Intervention duration subgroups.

There were overall subgroup differences by duration of intervention for waist circumference, fasting glucose or fasting insulin (all *P* < 0.05). Within subgroups, lifestyle interventions lasting for one to three months had greater reductions in, fasting glucose and fasting insulin compared to minimal treatment MD −0.25 mmol/L (95% CI [−0.43, −0.07] *p* < 0.01) and MD −7.03 pmol/L (95% CI [−12.47, −1.59] *p* < 0.01), respectively (Table 4). Interventions lasting more than three months were effective in reducing total testosterone levels MD −0.18nmol/L (95% CI [−0.36, −0.01] *p* = 0.04). There were no significant differences by duration of lifestyle intervention for all other measured outcomes (Table 4, S5 Appendix).

**Table 4:** Summary of effectiveness by duration of interventions.

Outcome	Subgroup	Studies	Participants	Mean Difference (95% CI)	*P*-value	*I*^2^ (%)
Weight (kg)	Medium term	11	536	−3.48. [−7.50, 0.54]	0.09	94
	Long term	10	529	−0.43 [−1.47, 0.60]	0.41	0
BMI	Medium term	15	720	−0.47 [−1.67, 0.73]	0.44	84
	Long term	13	599	−0.18 [−0.47, 0.11]	0.23	0
Waist circumference (cm)	Medium term	10	518	−3.11 [−6.48, 0.27]	0.07	90
	Long term	8	397	−1.66 [−4.59, 1.27]	0.27	80
Waist-hip ratio	Medium term	8	403	−0.02 [−0.06, 0.01]	0.22	91
	Long term	6	308	−0.01 [−0.05, 0.03]	0.74	75
Sex hormone-binding globulin (nmol/L)	Medium term	6	317	5.18 [0.33, 10.02]	0.04	63
	Long term	8	399	0.14 [−2.44, 2.72]	0.92	0
Total testosterone (nmol/L)	Medium term	11	548	0.00 [−0.30, 0.30]	0.98	86
	**Long term**	**8**	**434**	**−0.18 [−0.36, −0.01]**	**0.04**	**17**
Free Androgen Index	Medium term	4	161	−0.45 [−1.42, 0514]	0.36	0
	Long term	8	399	−0.44 [−1.36, 0.49]	0.35	56
Fasting glucose (mmol/L)	**Medium term**	**11**	**522**	**−0.25 [−0.43, −0.07]**	<0.01	85
	Long term	11	523	−0.02 [−0.21, 0.17]	0.85	87
Fasting insulin (pmol/L)	**Medium term**	**9**	**431**	**−7.03 [−12.47, −1.59.]**	**0.01**	**86**
	Long term	9	393	−3.06 [−7.03, 0.91]	0.13	4
Total cholesterol (mmol/L)	Medium term	7	298	−0.11 [−0.30, 0.08]	0.26	45
	Long term	10	449	−0.07 [−0.22, 0.08]	0.37	36
High-density lipoprotein cholesterol (mmol/L)	Medium term	5	217	0.02 [−0.06, 0.11]	0.58	43
	Long term	10	449	0.00 [0.05, 0.04]	0.88	23
Low-density lipoprotein cholesterol	Medium term	6	276	−0.18 [−0.41, 0.05]	0.12	72
	Long term	9	365	−0.09 [−0.24, 0.06]	0.25	27
Triglycerides (mmol/L)	Medium term	7	298	0.12 [−0.03, 0.26]	0.11	61
	Long term	9	409	0.04 [−0.8, 0.15]	0.54	9

#### Baseline BMI status subgroups.

**Fig 4:**
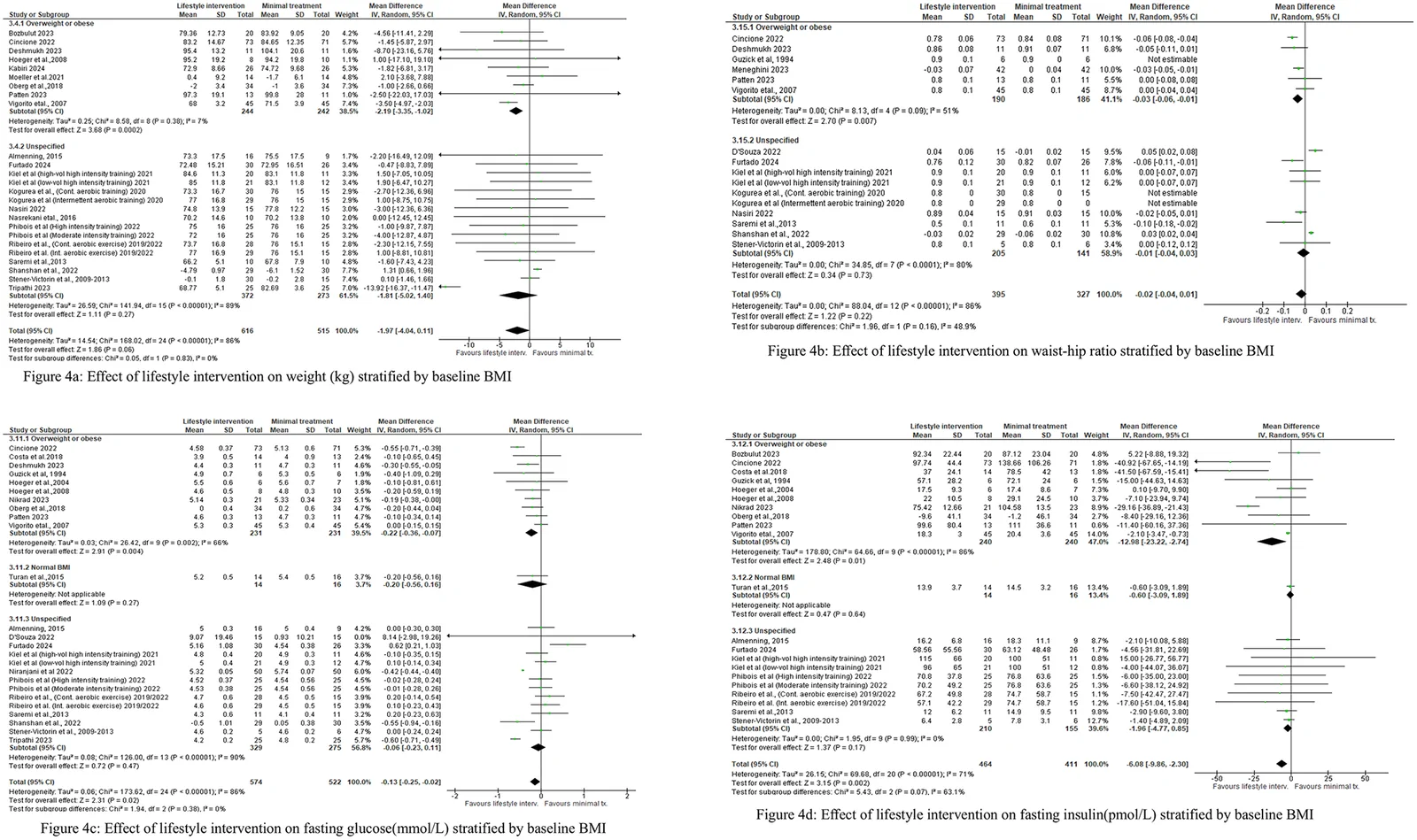
Effect of lifestyle intervention on weight, waist-hip ratio, fasting glucose, and fasting insulin stratified by baseline BMI.

**Fig 5:**
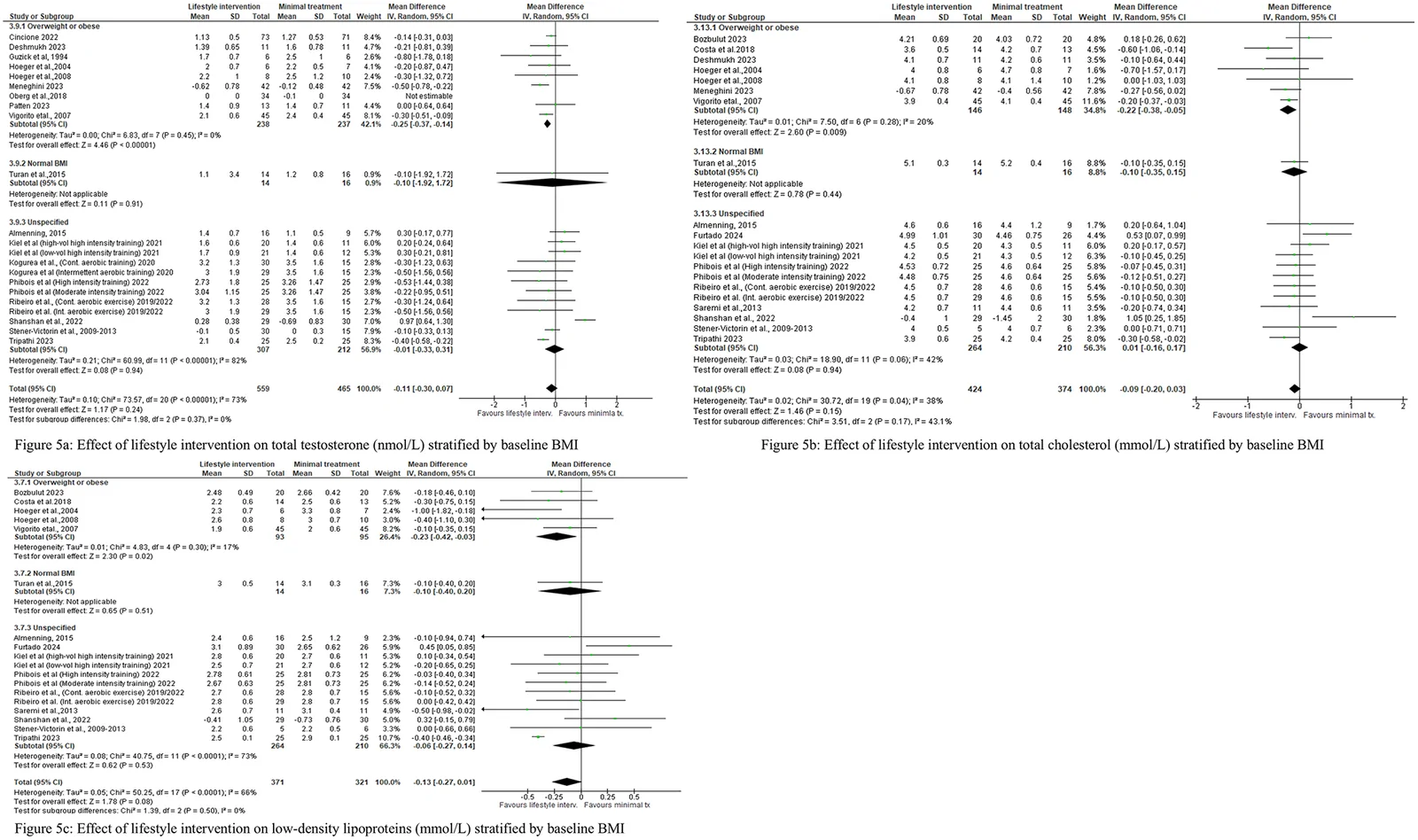
Effect of lifestyle intervention on total testosterone, total cholesterol, and low-density lipoproteins stratified by baseline BMI.

Lifestyle interventions in the overweight or obese subgroup demonstrated greater reductions in weight MD −2.19 kg (95% CI [−3.35, −1.02] *p* = 0.004, Fig 4a), waist-hip ratio MD −0.03 (95% CI [−0.06, −0.01] *p* = 0.007, Fig 4b), fasting glucose MD −0.22 mmol/L (95% CI [−0.36, −0.07] *p* = 0.004, Fig 4c), fasting insulin MD −12.98 pmol/L (95% CI [−23.22, −2.74] *p* = 0.01, Fig 4d), total testosterone MD −0.25 nmol/L (95% CI [−0.37, −0.14] *p* < 0.00001, Fig 5a), total cholesterol MD −0.22 mmol/L (95% CI [−0.38, −0.05] *p* = 0.09, Fig 5b), and low-density lipoproteins MD −0.23 mmol/L (95% CI [−0.42, −0.03] *p* = 0.02, Fig 5c) compared with minimal treatment. Within subgroups with normal or unspecified baseline BMI, there were no differences in any outcomes between lifestyle interventions and minimal treatment (Table 5, S2 Appendix).

**Table 5:** Summary of effectiveness of lifestyle intervention by baseline participants BMI.

Outcome	Subgroup	Studies	Participants	Mean Difference (95% CI)	*I*^2^ (%)
Weight (kg)	**Overweight or obese**	**9**	**486**	**−2.19 [−3.35, −1.02]**	**7**
	Normal BMI	0	0	–	–
	Unspecified	12	579	−1.81 [−5.02, 1.40]	89
BMI	Overweight or obese	12	608	−0.47 [−1.66, 0.73]	73
	Normal BMI	1	30	0.00 [−3.04, 3.04]	–
	Unspecified	15	679	−0.47 [−1.14, 0.20]	70
Waist circumference (cm)	Overweight or obese	9	501	−2.70 [−7.25, 1.85]	88
	Normal BMI	1	30	−1.00 [−9.68, 7.68]	–
	Unspecified	8	384	−2.31 [−5.43, 0.81]	92
Waist-hip ratio	**Overweight or obese**	**6**	**376**	**−0.03 [−0.06, −0.01]**	**51**
	Normal BMI	0	0	–	–
	Unspecified	8	320	−0.01 [−0.04, 0.03]	80
Sex hormone-binding globulin (nmol/L)	Overweight or obese	8	391	3.16 [0.33, 5.99]	43
	Normal BMI	0	0	–	–
	Unspecified	6	325	−0.54 [−8.01, 6.94]	23
Total testosterone (nmol/L)	**Overweight or obese**	**9**	**475**	**−0.25 [−0.37, −0.14]**	**0**
	Normal BMI	1	30	−0.10 [−1.92, 1.72]	–
	Unspecified	9	477	−0.01 [−0.33, 0.31]	82
Free Androgen Index	Overweight or obese	6	235	−0.54 [−2.06, 0.99]	51
	Normal BMI	0	0	–	–
	Unspecified	6	325	−0.56 [−1.32, 0.19]	39
Fasting glucose (mmol/L)	Overweight or obese	10	452	−0.22 [−0.36, −0.07]	66
	Normal BMI	1	30	−0.20 [−0.56, 0.16]	–
	Unspecified	11	553	−0.06 [−0.23, 0.11]	90
Fasting insulin (pmol/L)	**Overweight or obese**	**10**	**480**	**−12.98 [−23.22, −2.74]**	**86**
	Normal BMI	1	30	−0.60 [−3.09, 1.89]	–
	Unspecified	7	314	−1.96 [−4.77, 0.85]	0
Total cholesterol (mmol/L)	**Overweight or obese**	**7**	**294**	**−0.22 [−0.38, −0.05]**	**20**
	Normal BMI	1	30	−0.10 [−0.35, 0.15]	–
	Unspecified	9	423	0.01 [−0.16, 0.17]	42
High-density lipoprotein cholesterol (mmol/L)	Overweight or obese	6	272	−0.01 [−0.06, 0.05]	19
	Normal BMI	1	30	0.00 [−0.07, 0.07]	–
	Unspecified	8	364	0.01 [−0.06, 0.08]	39
Low-density lipoprotein cholesterol (mmol/L)	**Overweight or obese**	**5**	**188**	**−0.23 [−0.42, −0.03]**	**17**
	Normal BMI	1	30	−0.10 [−0.40, 0.20]	–
	Unspecified	9	423	−0.06 [−0.27, 0.14]	73
Triglycerides (mmol/L)	Overweight or obese	6	254	0.00 [−0.19, 0.19]	49
	Normal BMI	1	30	–	–
	Unspecified	9	423	0.10 [−0.00, 0.20]	21

There were no significant subgroup differences by baseline BMI status (overweight or obese versus normal versus unspecified BMI) for any of the other outcomes assessed (Table 5, S5 Appendix).

## Discussion

This systematic review and meta-analysis provide quantitative estimates of the effects of lifestyle interventions on reproductive, anthropometric and metabolic outcomes in women with PMOS. Overall, lifestyle intervention led to more than 4-fold increase in the odds of achieving regular menstrual cycles. Significant reductions in waist circumference, fasting glucose and fasting insulin were also observed when compared with minimal treatment. Subgroup analyses revealed variation in effect by intervention type, duration, and baseline BMI status, with beneficial effects on other outcomes seen within certain subgroups. Exercise-based interventions improved anthropometric outcomes and fasting insulin compared with minimal treatment. Similarly, lifestyle interventions lasting one to three months led to significant reductions in fasting glucose and fasting insulin, while interventions lasting more than three months improved total testosterone level. Among the subgroup of participants with baseline overweight or obesity, lifestyle intervention improved weight, waist-hip ratio, total testosterone, and lipid profile compared with minimal treatment.

The main strength of this review is the robust methodology, incorporation of the most recent evidence to date and building on prior systematic reviews. With the inclusion of 34 studies, making it the most comprehensive analysis to date on the impact of diet, exercise, and behaviour changes on a range of outcomes in PMOS. This review expands on current knowledge and improves generalisability of the findings by including studies from high-, upper-middle-, and lower-middle-income countries.

The main limitation of this review was that over a third of the included studies had overall high risk of bias, reflecting methodological weaknesses which may lead to misrepresentation of the true effects of lifestyle interventions. To mitigate this, we conducted sensitivity analysis by excluding these studies to establish their influence on the findings. Another limitation is the broad definition of lifestyle interventions, which encompass a wide variety of diet, exercise, behavioural, or combined interventions and contribute to heterogeneity in our findings. In addition, we included studies that used different PMOS diagnostic criteria (NIH 1990, Rotterdam 2003, and AES‑PCOS 2006), which may have resulted in variation in baseline phenotype and outcome responses across trials and represent a further important source of clinical heterogeneity. Due to substantial statistical heterogeneity across most outcomes, we did not formally assess publication bias using funnel plots or Egger’s test as they may be difficult to interpret in these conditions. Participant characteristics and baseline risk profiles can also differ and affect intervention responses. We addressed this to some extent with the inclusion of subgroup analyses by type and duration of lifestyle intervention and by baseline BMI categories of study participants. However, while these sub-analyses can provide valuable insights into the differential effects caused by these factors, statistical power is limited, particularly for some subgroups with small numbers of studies. Lastly, although many of the included studies adjusted for confounders, the variability in intervention protocols and outcome measurements across the included studies may have introduced additional confounders such as cultural dietary patterns and variations in adherence to interventions which are difficult to standardise across studies and regions. In addition, a formal assessment of study integrity was not undertaken because this review focussed on trial-level risk of bias and evidence certainty using the Cochrane RoB 2 tool and GRADE.

Improving on a previous review [1] which identified only two studies reporting on reproductive outcomes, we included five studies reporting on the effects of lifestyle interventions on menstrual regularity; three studies assessed menstrual regularity at the end of the study without specifying the exact time to cycle normalisation, one study reported regular menses at 90–120 days, and one study assessed this outcome by 6 months. Overall, we found lifestyle interventions to be associated with an over 4-fold increase in the odds of achieving regular cycles. Of the five studies providing data for this analysis, three [24,30,47] included participants with baseline overweight or obesity while the baseline BMI of the participants in the remaining two [26,56] were unspecified. This finding aligns with that of another systematic review of two studies involving women with PMOS and obesity, which found similarly improved menstrual cycles with lifestyle intervention OR 4.34 (95% CI [1.75, 10.78] *p* = 0.02) [59]. This substantial improvement in ovulatory function offers women with PMOS a compelling non-pharmacological first-line option, particularly reassuring given widespread fertility concerns. Unlike pharmacological treatments (combined oral contraceptives, insulin sensitizers, anti-androgens) carrying adverse effect risks, lifestyle interventions demonstrated reproductive benefits without medications, with three of five studies specifically including overweight/obese participants where PMOS cardiometabolic risk is highest [59].

Previous reviews have reported different effects of lifestyle intervention on weight loss in women with PMOS, likely due to differences in the inclusion criteria, intervention types and duration in the included studies. The 2019 Cochrane review involving nine studies and 353 women suggested that there was low-quality evidence that lifestyle intervention leads to better weight loss compared to minimal treatment MD −1.68 kg (95% CI [−2.66, −0.70]) [14]. This finding aligns with another systematic review that also reported beneficial effects of lifestyle interventions on weight management in women with PMOS and obesity, although that review was based on only three studies [59]. More recent syntheses, including a 2025 review of randomised controlled trials published in the last 10 years [60] and another review of observational and interventional studies identified in 2022 [16] have also generally supported modest improvements in weight. Our review did not find a statistically significant overall effect of lifestyle interventions on weight compared to minimal treatment which is consistent with findings of our previous review MD −1.02 (95% CI [−2.08, 0.04] *p* = 0.058) [17].

Importantly, subgroup analysis restricted to women with overweight or obesity found a significant reduction in weight with lifestyle interventions, suggesting that the effects are more pronounced in this population. These findings highlight the need to consider the impact of participant characteristics, study design, intervention type and duration when interpreting available evidence. Exercise, particularly moderate-to-vigorous aerobic exercise and combined aerobic-resistance training, most effectively reduces waist circumference and visceral adipose tissue in overweight and obese adults, producing clinically meaningful improvements in metabolic outcomes including insulin sensitivity, glycaemic control, lipid profiles, and overall cardiometabolic risk. These reductions occur, in part, through preferential lipolysis and oxidation of visceral fat, driven by its heightened responsiveness to exercise-induced catecholamines and myokines (such as IL-6) relative to subcutaneous depots. Higher-intensity protocols often amplify this effect in a dose-dependent manner [61].

The earlier review reported that lifestyle interventions improved waist circumference MD −1.32 cm (95% CI [−2.46, −0.18] 12 studies), waist/hip ratio MD −0.03 (95% CI [−0.05, −0.01] 6 studies), total cholesterol MD −0.15 mmol/L (95% CI [−0.26, −0.03] 12 studies) and low density lipoprotein cholesterol MD −0.15 mmol/L (95% CI [−0.28, −0.02] 12 studies) compared to minimal treatment in women with PMOS [1]. In this review, the significant beneficial effects of lifestyle interventions on waist circumference were corroborated in a larger analysis involving pooled data from 17 studies. However, the significant effects on waist/hip ratio total cholesterol and low-density lipoprotein cholesterol were only observed among the subgroup of women with baseline overweight or obesity in subgroup analysis. Reductions in waist circumference, fasting glucose and fasting insulin translate to clinically meaningful improvements in central adiposity and insulin resistance, key drivers of PMOS cardiometabolic risk.

While the 2019 Cochrane review found that lifestyle interventions may improve free androgen index (MD −1.11 (95% CI [−1.96, −0.26] 6 studies, 204 women, *I*^2^ = 71%, low-quality evidence)) [14], we found no significant overall effect on free androgen index or total testosterone. However, subgroup analyses demonstrate greater reductions in total testosterone (MD −0.25 nmol/L (95% CI [−0.37, −0.14] 9 studies, 475 women)) specifically among women with baseline overweight or obesity, indicating particular benefit for this population. Similarly, while the Cochrane review found lifestyle interventions to be effective in reducing total cholesterol (MD −0.14 mmol (95% CI [−0.25, −0.02] 9 studies, 331 women)) and low-density lipoprotein levels (MD −0.16 mmol (95% CI [−0.29, −0.03] 9 studies, 326 women)) [14], we found statistically significant beneficial effects only in the subgroup of participants with baseline overweight or obesity.

In our pooled analysis of 18 studies, lifestyle intervention significantly reduced fasting insulin levels. This finding aligns with, and expand on that of the earlier reviews that included 14 and 10 studies, which also reported significant reductions in fasting insulin MD of −1.8 pmol/L (95% CI [−3.10, −0.65]) [1] and MD −1.42 µU/mL (95% CI [−2.44, −0.39]) [14], respectively. In both the earlier review [1] and the 2019 Cochrane review [14], there were no differences in fasting glucose levels between the lifestyle intervention and minimal treatment groups in pooled analyses of 15 and 11 studies, respectively. With 22 included studies, we found that lifestyle intervention led to significant reductions in fasting glucose levels; however, this was not corroborated in a sensitivity analysis where studies with high risk of bias were excluded, suggesting caution in interpretation of this finding.

The findings of this review reinforce the importance of lifestyle interventions as a first-line approach for managing symptomatic women with PMOS. The observed improvements in menstrual regularity, central adiposity, fasting glucose and fasting insulin levels associated with lifestyle interventions highlight the potential benefits of incorporating these approaches as essential components of clinical PMOS care. These results support the 2023 International Evidence-based Guideline for the Assessment and Management of PCOS, which recommends lifestyle intervention as the initial step in management [1]. Clinicians should prioritise exercise-based programs for improving central adiposity and insulin resistance, and dietary interventions for glycaemic control, particularly in those with overweight or obesity where cardiometabolic benefits are most pronounced. Implementation requires multidisciplinary support (e.g., dietitians, physiotherapists) and digital tools for adherence, with shared decision-making to tailor strategies to patient preferences and barriers.

Our analyses indicate that different lifestyle interventions may be more effective for specific outcomes or population subgroups, underscoring the importance of personalised care. Diet modifications—encompassing a heterogeneous range of dietary approaches are suggested to be more appropriate in managing glucose levels, while exercise interventions, alongside which patients often adopted modest dietary restriction may be better suited for improving BMI and waist circumference and reducing insulin levels. Additionally, among PMOS subgroups with overweight and obesity, lifestyle interventions improved weight, fasting glucose, insulin, testosterone, and dyslipidaemia, suggesting particular benefit in this subgroup for managing cardiometabolic risk.

Taken together, these findings emphasise the need for future research into the mechanisms through which specific lifestyle strategies impact reproductive, anthropometric, and metabolic parameters in PMOS. Notably, 78% of the included studies in this review were assessed as having some concerns or high risk of bias. This underscores the need for better-designed and implemented trials, prioritise methodological rigour, including appropriate randomisation, blinding of treatment allocation, and standardised outcome reporting. Additional research is also needed to establish the effects of lifestyle interventions across the Rotterdam PCOS phenotypes (A: hyperandrogenism + ovulatory dysfunction + polycystic ovarian morphology; B: hyperandrogenism + ovulatory dysfunction; C: hyperandrogenism + polycystic ovarian morphology; D: ovulatory dysfunction + polycystic ovarian morphology), as responses may vary depending on underlying pathophysiology [19,62,63]. A more nuanced understanding of these differences can support the development of tailored and evidence-based approaches to care. Lifestyle interventions significantly improved menstrual regularity and reduced waist circumference, fasting glucose and insulin levels compared to minimal treatment, reinforcing their role as a first-line approach in PMOS management. These findings emphasise the importance of personalised care, with tailored lifestyle interventions to address the diverse needs of women with PMOS. Further research is needed to identify the most effective intervention components and combinations for specific outcomes, and their impacts on fertility and broader outcomes including psychological features in PMOS.

## Supporting information

S1 Checklist PRISMA checklist for reporting systematic reviews and meta-analyses. Adapted from Page MJ, McKenzie JE, Bossuyt PM, Boutron I, Hoffmann TC, Mulrow CD, et al. The original checklist is available at: https://www.prisma-statement.org/.
(DOCX)

S1 Appendix Search terms and strategy. (DOCX)

S2 Appendix Characteristics of included studies. (DOCX)

S3 Appendix Risk of bias assessment. (DOCX)

S4 Appendix GRADE assessment. (DOCX)

S5 Appendix Forest plots of individual analyses. (DOCX)

## Abbreviations

BMI: body mass index
CI: confidence intervals
GRADE: Grading of Recommendations Assessment, Development and Evaluation
MD: mean differences
OR: odd ratios
PCOS: replaces polycystic ovary syndrome
PMOS: polyendocrine metabolic ovarian syndrome
PRISMA: Preferred Reporting Items for Systematic Reviews and Meta-analyses
RCTs: randomised controlled trials
ROB: Risk of Bias
SD: standard deviations

## References

[1] Teede H, Tay CT, Laven J. et al. International evidence-based guideline for the assessment and management of polycystic ovary syndrome. Monash University; 2023. doi: 10.26180/24003834.v1

[2] Parker J, O’Brien C, Hawrelak J, Gersh FL. Polycystic ovary syndrome: an evolutionary adaptation to lifestyle and the environment. Int J Environ Res Public Health. 2022;19(3):1336. doi: 10.3390/ijerph19031336 35162359 PMC8835454

[3] Teede H, Deeks A, Moran L. Polycystic ovary syndrome: a complex condition with psychological, reproductive and metabolic manifestations that impacts on health across the lifespan. BMC Med. 2010;8:41. doi: 10.1186/1741-7015-8-41 20591140 PMC2909929

[4] Sirmans S, Pate K. Epidemiology, diagnosis, and management of polycystic ovary syndrome. Clin Epidemiol 2013:1. doi: 10.2147/CLEP.S37559 PMC3872139 24379699

[5] Bahri Khomami M, Hashemi S, Shorakae S, Harrison CL, Piltonen TT, Romualdi D, et al. Systematic review and meta-analysis of birth outcomes in women with polycystic ovary syndrome. Nat Commun. 2024;15(1):5592. doi: 10.1038/s41467-024-49752-6 38965241 PMC11224419

[6] Teede HJ, Khomami MB, Morman R, Laven JSE, Joham AE, Costello MF, et al. Polyendocrine metabolic ovarian syndrome, the new name for polycystic ovary syndrome: a multistep global consensus process. Lancet. 2026;407(10545):2329–39. doi: 10.1016/S0140-6736(26)00717-8 42119588

[7] Mykhalchenko K, Lizneva D, Trofimova T, Walker W, Suturina L, Diamond MP, et al. Genetics of polycystic ovary syndrome. Expert Rev Mol Diagn. 2017;17(7):723–33. doi: 10.1080/14737159.2017.1340833 28602111

[8] Apridonidze T, Essah PA, Iuorno MJ, Nestler JE. Prevalence and characteristics of the metabolic syndrome in women with polycystic ovary syndrome. J Clin Endocrinol Metab. 2005;90(4):1929–35. doi: 10.1210/jc.2004-1045 15623819

[9] Chen W, Pang Y. Metabolic Syndrome and PCOS: pathogenesis and the role of metabolites. Metabolites. 2021;11(12):869. doi: 10.3390/metabo11120869 34940628 PMC8709086

[10] Tay CT, Mousa A, Vyas A, Pattuwage L, Tehrani FR, Teede H. 2023 international evidence-based polycystic ovary syndrome guideline update: insights from a systematic review and meta-analysis on elevated clinical cardiovascular disease in polycystic ovary syndrome. J Am Heart Assoc. 2024;13(16):e033572. doi: 10.1161/JAHA.123.033572 39119982 PMC11963914

[11] Teede HJ, Hutchison SK, Zoungas S. The management of insulin resistance in polycystic ovary syndrome. Trends Endocrinol Metab. 2007;18(7):273–9. doi: 10.1016/j.tem.2007.08.001 17698366

[12] Diamanti-Kandarakis E, Papavassiliou AG. Molecular mechanisms of insulin resistance in polycystic ovary syndrome. Trends Mol Med. 2006;12(7):324–32. doi: 10.1016/j.molmed.2006.05.006 16769248

[13] Stepto NK, Cassar S, Joham AE, Hutchison SK, Harrison CL, Goldstein RF, et al. Women with polycystic ovary syndrome have intrinsic insulin resistance on euglycaemic-hyperinsulaemic clamp. Hum Reprod. 2013;28(3):777–84. doi: 10.1093/humrep/des463 23315061

[14] Lim SS, Hutchison SK, Van Ryswyk E, Norman RJ, Teede HJ, Moran LJ. Lifestyle changes in women with polycystic ovary syndrome. Cochrane Database Syst Rev. 2019;3(3):CD007506. doi: 10.1002/14651858.CD007506.pub4 30921477 PMC6438659

[15] Long JR, Parker M, Jumani S, Ahmed A, Huynh V, Gomez-Lobo V. Effect of lifestyle modifications on polycystic ovary syndrome in predominantly young adults: a systematic review. J Pediatr Adolesc Gynecol. 2025;38(2):139-147.e4. doi: 10.1016/j.jpag.2024.11.003 39577757 PMC11875956

[16] Mohamed AH, Albasheer O, Ghoniem MA, Abdalghani N, Ayish F, Abdelwahab SI, et al. Impact of lifestyle interventions on reproductive and psychological outcomes in women with polycystic ovary syndrome: a systematic review. Medicine (Baltimore). 2025;104(3):e41178. doi: 10.1097/MD.0000000000041178 39833063 PMC11749783

[17] Mousa A, Tay CT, Teede H. Technical report for the 2023 international evidence-based guideline for the assessment and management of polycystic ovary syndrome. Monash University; 2023. doi: 10.26180/23625288.v1

[18] Page MJ, McKenzie JE, Bossuyt PM, Boutron I, Hoffmann TC, Mulrow CD, et al. The PRISMA 2020 statement: an updated guideline for reporting systematic reviews. Rev Esp Cardiol (Engl Ed). 2021;74(9):790–9. doi: 10.1016/j.rec.2021.07.010 34446261

[19] Rotterdam ESHRE/ASRM-Sponsored PCOS Consensus Workshop Group. Revised 2003 consensus on diagnostic criteria and long-term health risks related to polycystic ovary syndrome. Fertil Steril. 2004;81(1):19–25. doi: 10.1016/j.fertnstert.2003.10.004 14711538

[20] Azziz R, Carmina E, Dewailly D, Diamanti-Kandarakis E, Escobar-Morreale HF, Futterweit W, et al. Positions statement: criteria for defining polycystic ovary syndrome as a predominantly hyperandrogenic syndrome: an Androgen Excess Society guideline. J Clin Endocrinol Metab. 2006;91(11):4237–45. doi: 10.1210/jc.2006-0178 16940456

[21] Al Wattar BH, Teede H, Garad R, Franks S, Balen A, Bhide P, et al. Harmonising research outcomes for polycystic ovary syndrome: an international multi-stakeholder core outcome set. Hum Reprod. 2020;35(2):404–12. doi: 10.1093/humrep/dez272 32020203

[22] Sterne JAC, Savović J, Page MJ, Elbers RG, Blencowe NS, Boutron I, et al. RoB 2: a revised tool for assessing risk of bias in randomised trials. BMJ. 2019;:l4898. doi: 10.1136/bmj.l4898 31462531

[23] Review Manager (RevMan) [Internet]. Version 5. The Cochrane Collaboration; 2024 [cited 2024 Sep 8]. Available from: https://revman.cochrane.org/info

[24] Patten RK, McIlvenna LC, Levinger I, Garnham AP, Shorakae S, Parker AG, et al. High-intensity training elicits greater improvements in cardio-metabolic and reproductive outcomes than moderate-intensity training in women with polycystic ovary syndrome: a randomized clinical trial. Hum Reprod. 2022;37(5):1018–29. doi: 10.1093/humrep/deac047 35325125

[25] Kiel IA, Lionett S, Parr EB, Jones H, Røset MAH, Salvesen Ø. 1 High-intensity interval training in polycystic ovary syndrome: a two-centre, three-armed 2 randomized controlled trial. n.d. 10.1249/MSS.0000000000002849 35019901

[26] Mei S, Ding J, Wang K, Ni Z, Yu J. Mediterranean diet combined with a low-carbohydrate dietary pattern in the treatment of overweight polycystic ovary syndrome patients. Front Nutr. 2022;9:876620. doi: 10.3389/fnut.2022.876620 35445067 PMC9014200

[27] Moeller LV, Lindhardt CL, Andersen MS, Glintborg D, Ravn P. Motivational interviewing in obese women with polycystic ovary syndrome - a pilot study. Gynecol Endocrinol. 2019;35(1):76–80. doi: 10.1080/09513590.2018.1498832 30182773

[28] Stefanaki C, Bacopoulou F, Livadas S, Kandaraki A, Karachalios A, Chrousos GP, et al. Impact of a mindfulness stress management program on stress, anxiety, depression and quality of life in women with polycystic ovary syndrome: a randomized controlled trial. Stress. 2015;18(1):57–66. doi: 10.3109/10253890.2014.974030 25287137

[29] Vigorito C, Giallauria F, Palomba S, Cascella T, Manguso F, Lucci R, et al. Beneficial effects of a three-month structured exercise training program on cardiopulmonary functional capacity in young women with polycystic ovary syndrome. J Clin Endocrinol Metab. 2007;92(4):1379–84. doi: 10.1210/jc.2006-2794 17264174

[30] Meneghini C, Bianco C, Galanti F, Tamburelli V, Dal Lago A, Licata E, et al. The impact of nutritional therapy in the management of overweight/obese PCOS patient candidates for IVF. Nutrients. 2023;15(20):4444. doi: 10.3390/nu15204444 37892519 PMC10609803

[31] Cincione IR, Graziadio C, Marino F, Vetrani C, Losavio F, Savastano S, et al. Short-time effects of ketogenic diet or modestly hypocaloric Mediterranean diet on overweight and obese women with polycystic ovary syndrome. J Endocrinol Invest. 2023;46(4):769–77. doi: 10.1007/s40618-022-01943-y 36401759

[32] Dietz de Loos A, Jiskoot G, Beerthuizen A, Busschbach J, Laven J. Metabolic health during a randomized controlled lifestyle intervention in women with PCOS. Eur J Endocrinol. 2021;186(1):53–64. doi: 10.1530/EJE-21-0669 34714771 PMC8679850

[33] Almenning I, Rieber-Mohn A, Lundgren KM, Shetelig Løvvik T, Garnæs KK, Moholdt T. Effects of high intensity interval training and strength training on metabolic, cardiovascular and hormonal outcomes in women with polycystic ovary syndrome: a pilot study. PLoS One. 2015;10(9):e0138793. doi: 10.1371/journal.pone.0138793 26406234 PMC4583183

[34] Stener-Victorin E, Holm G, Janson PO, Gustafson D, Waern M. Acupuncture and physical exercise for affective symptoms and health-related quality of life in polycystic ovary syndrome: secondary analysis from a randomized controlled trial. BMC Complement Altern Med. 2013;13:131. doi: 10.1186/1472-6882-13-131 23763822 PMC3684530

[35] Oberg E, Gidlöf S, Jakson I, Mitsell M, Tollet Egnell P, Hirschberg AL. Improved menstrual function in obese women with polycystic ovary syndrome after behavioural modification intervention: a randomized controlled trial. Clin Endocrinol (Oxf). 2019;90(3):468–78. doi: 10.1111/cen.13919 30565716

[36] Deshmukh H, Papageorgiou M, Wells L, Akbar S, Strudwick T, Deshmukh K, et al. The effect of a Very-Low-Calorie Diet (VLCD) vs. a moderate energy deficit diet in obese women with Polycystic Ovary Syndrome (PCOS): a randomised controlled trial. Nutrients. 2023;15(18):3872. doi: 10.3390/nu15183872 37764656 PMC10536436

[37] Brown AJ, Setji TL, Sanders LL, Lowry KP, Otvos JD, Kraus WE, et al. Effects of exercise on lipoprotein particles in women with polycystic ovary syndrome. Med Sci Sports Exerc. 2009;41(3):497–504. doi: 10.1249/MSS.0b013e31818c6c0c 19204602 PMC2727938

[38] Guzick DS, Wing R, Smith D, Berga SL, Winters SJ. Endocrine consequences of weight loss in obese, hyperandrogenic, anovulatory women. Fertil Steril. 1994;61(4):598–604. doi: 10.1016/s0015-0282(16)56632-1 8150098

[39] Hoeger KM, Kochman L, Wixom N, Craig K, Miller RK, Guzick DS. A randomized, 48-week, placebo-controlled trial of intensive lifestyle modification and/or metformin therapy in overweight women with polycystic ovary syndrome: a pilot study. Fertil Steril. 2004;82(2):421–9. doi: 10.1016/j.fertnstert.2004.02.104 15302293

[40] Hoeger K, Davidson K, Kochman L, Cherry T, Kopin L, Guzick DS. The impact of metformin, oral contraceptives, and lifestyle modification on polycystic ovary syndrome in obese adolescent women in two randomized, placebo-controlled clinical trials. J Clin Endocrinol Metab. 2008;93(11):4299–306. doi: 10.1210/jc.2008-0461 18728175 PMC2582568

[41] Costa EC, DE Sá JCF, Stepto NK, Costa IBB, Farias-Junior LF, Moreira SDNT, et al. Aerobic training improves quality of life in women with polycystic ovary syndrome. Med Sci Sports Exerc. 2018;50(7):1357–66. doi: 10.1249/MSS.0000000000001579 29443823

[42] Kogure GS, Lopes IP, Ribeiro VB, Mendes MC, Kodato S, Furtado CLM, et al. The effects of aerobic physical exercises on body image among women with polycystic ovary syndrome. J Affect Disord. 2020;262:350–8. doi: 10.1016/j.jad.2019.11.025 31735408

[43] Ribeiro VB, Lopes IP, Dos Reis RM, Silva RC, Mendes MC, Melo AS, et al. Continuous versus intermittent aerobic exercise in the improvement of quality of life for women with polycystic ovary syndrome: a randomized controlled trial. J Health Psychol. 2021;26(9):1307–17. doi: 10.1177/1359105319869806 31495231

[44] Miranda Furtado CL, Hansen M, Kogure GS, Ribeiro VB, Taylor N, Racy Soares M, et al. Resistance and aerobic training increases genome-wide DNA methylation in women with polycystic ovary syndrome. Epigenetics. 2024;19(1):2305082. doi: 10.1080/15592294.2024.2305082 38245873 PMC10802204

[45] Philbois SV, Ribeiro VB, Tank J, Dos Reis RM, Gerlach DA, Souza HCD. Cardiovascular autonomic modulation differences between moderate-intensity continuous and high-intensity interval aerobic training in women with PCOS: a randomized trial. Front Endocrinol (Lausanne). 2022;13:1024844. doi: 10.3389/fendo.2022.1024844 36568110 PMC9782449

[46] Colonetti L, Uggioni MLR, Prestes G da S, Stangherlin L, Junior JCD, Moura R, et al. Effects of carbohydrate reduced diet associated with strength training on clinical signs of women with polycystic ovary syndrome: randomized clinical trial. Nutrition. 2025;133:112696. doi: 10.1016/j.nut.2025.112696 40048764

[47] Bozbulut R, Döğer E, Çamurdan MO, Bideci A. Beneficial effects of RESMENA diet on anthropometric, metabolic, and reproductive profile in adolescents with obesity and polycystic ovary syndrome: a randomized controlled intervention study. Horm Res Paediatr. 2024;97(5):483–95. doi: 10.1159/000535053 37952514 PMC11446335

[48] Turan V, Mutlu EK, Solmaz U, Ekin A, Tosun O, Tosun G, et al. Benefits of short-term structured exercise in non-overweight women with polycystic ovary syndrome: a prospective randomized controlled study. J Phys Ther Sci. 2015;27(7):2293–7. doi: 10.1589/jpts.27.2293 26311969 PMC4540866

[49] Nasrekani Z, Fathi M. The effect of 12 weeks aerobic training on body composition, aerobic power and some hormones in infertile women with polycystic ovary syndrome. Iran J Obstet Gynecol Infertil. 2016;19(5):1–10. (Persian).

[50] Saremi A, Shoundi N, Kermani M, Kazemi M. Serum anti-Müllerian hormone levels after exercise training in women with polycystic ovary syndrome: a randomized clinical trial. Iran J Obstet Gynecol Infertil. 2013;16(64):10–8. (Persian).

[51] Nikrad N, Farhangi MA, Fard Tabrizi FP, Vaezi M, Mahmoudpour A, Mesgari-Abbasi M. The effect of calorie-restriction along with thylakoid membranes of spinach on the gut-brain Axis Pathway and oxidative stress biomarkers in obese women with polycystic ovary syndrome: a Randomized, Double-blind, placebo-controlled clinical trial. J Ovarian Res. 2023;16(1):216. doi: 10.1186/s13048-023-01288-x 37968684 PMC10652637

[52] Nasiri M, Monazzami A, Alavimilani S, Asemi Z. The effect of high intensity intermittent and combined (resistant and endurance) trainings on some anthropometric indices and aerobic performance in women with polycystic ovary syndrome: a randomized controlled clinical trial study. Int J Fertil Steril. 2022;16(4):268–74. doi: 10.22074/ijfs.2022.551096.1279 36273312 PMC9627015

[53] Kabiri SS, Javanbakht Z, Zangeneh M, Moludi J, Saber A, Salimi Y, et al. The effects of MIND diet on depression, anxiety, quality of life and metabolic and hormonal status in obese or overweight women with polycystic ovary syndrome: a randomised clinical trial. Br J Nutr. 2026;135(5):496–509. doi: 10.1017/S0007114524001168 39465581

[54] Tripathi PP. Effect of wolly workout in PCOS and PCOD patients as part of management. SSR-IIJLS. 2023;9(4):3281–9. doi: 10.21276/ssr-iijls.2023.9.4.7

[55] Niranjani S, Bhuvaneswari G, Hemamalini M, Vijayalakshmi R. Efficacy of cinnamon, exercise and counselling (multi-interventional Package) on insulin resistance among young girls with Polycystic Ovarian Syndrome. J Pharm Negat Results. 2022;13(Special Issue 9):2463–8. doi: 10.47750/pnr.2022.13.S09.292

[56] Souza PD, Rodrigues DE, Kaipangala RG, Leena KC. Effectiveness of multimodular interventions of lifestyle modification on symptoms of polycystic ovarian syndrome and quality of life among women: a quasi-experimental study. J Clin Diagn Res 2022. doi: 10.7860/JCDR/2022/50394.16030

[57] Meenakshi M, Anitha A, Ramana K, Kamalakannan M. Efficacy of Swiss ball exercise and resistance training in polycystic ovarian syndrome. IJPOT. 2024;18:302–9. doi: 10.37506/71dzqn30

[58] Dietz de Loos A, Jiskoot G, Louwers Y, Beerthuizen A, Busschbach J, Laven J. Pregnancy outcomes in women with PCOS: follow-up study of a randomized controlled three-component lifestyle intervention. J Clin Med. 2023;12(2):426. doi: 10.3390/jcm12020426 36675355 PMC9867443

[59] Kim C-H, Lee S-H. Effectiveness of lifestyle modification in polycystic ovary syndrome patients with obesity: a systematic review and meta-analysis. Life (Basel). 2022;12(2):308. doi: 10.3390/life12020308 35207595 PMC8876590

[60] Gautam R, Maan P, Jyoti A, Kumar A, Malhotra N, Arora T. The role of lifestyle interventions in PCOS management: a systematic review. Nutrients. 2025;17(2):310. doi: 10.3390/nu17020310 39861440 PMC11767734

[61] Aerobic Exercise and Weight Loss in Adults: A Systematic Review and Dose-Response Meta-Analysis | Nutrition, Obesity, Exercise | JAMA Network Open | JAMA Network. n.d. [cited 2026 April 1]. Available from: https://jamanetwork.com/journals/jamanetworkopen/fullarticle/2828487 10.1001/jamanetworkopen.2024.52185 PMC11672165 39724371

[62] Guastella E, Longo RA, Carmina E. Clinical and endocrine characteristics of the main polycystic ovary syndrome phenotypes. Fertil Steril. 2010;94(6):2197–201. doi: 10.1016/j.fertnstert.2010.02.014 20303485

[63] Unfer V, Kandaraki E, Pkhaladze L, Roseff S, Vazquez-Levin MH, Laganà AS, et al. When one size does not fit all: reconsidering PCOS etiology, diagnosis, clinical subgroups, and subgroup-specific treatments. Endocr Metab Sci 2024;14:100159. doi: 10.1016/j.endmts.2024.100159

