# Supplementary material for: The effect of lifestyle interventions in women with polyendocrine metabolic ovarian syndrome: A systematic review and meta-analysis
Source: PLoS Med. 2026 Sep 23;23(9):e1004887. doi: 10.1371/journal.pmed.1004887 (PMC13600514; doi:10.1371/journal.pmed.1004887)
Supplement: Supplementary file 2 [file pmed.1004887.s002.docx]

S1 Appendix: Database searches

Embase

| **#** | **Searches** | **Results** |
| --- | --- | --- |
| 1 | ovary polycystic disease/ or exp *ovary cyst/ | 42671 |
| 2 | ovarian degeneration.mp. | 52 |
| 3 | sclerocystic ovarian syndrome$.mp. | 2 |
| 4 | sclerocystic ovarian degeneration.mp. | 3 |
| 5 | exp *ovary polycystic disease/ | 23333 |
| 6 | PCO.mp. | 5664 |
| 7 | 1 or 2 or 3 or 4 or 5 or 6 | 46884 |
| 8 | *contraception/ or *contraceptive agent/ or *hormonal contraceptive agent/ or *menstruation inducing agent/ or *oral contraceptive agent/ | 47067 |
| 9 | *metformin/ or metformin.mp. | 94814 |
| 10 | sex hormone anatagonist.mp. | 0 |
| 11 | *human/ or exp *antiandrogen therapy/ or spironolactone/ or *cyproterone acetate/ | 745329 |
| 12 | *antiandrogen/ or *sex hormone antagonist/ or *cyproterone/ or *cyproterone acetate/ or *flutamide/ or *spironolactone/ | 18589 |
| 13 | *finasteride/ | 2766 |
| 14 | *antiobesity agent/ | 3450 |
| 15 | orlistat.mp. or *tetrahydrolipstatin/ | 3922 |
| 16 | sibutramine.mp. or *sibutramine/ | 4874 |
| 17 | *inositol phosphate/ or *inositol polyphosphate/ or *inositol pentakisphosphate/ or *inositol/ or inositol trisphosphate/ or *inositol derivative/ or *inositol tetrakisphosphate/ | 11791 |
| 18 | *oral contraceptive agent/ | 18723 |
| 19 | clomifene/ or *fertility promoting agent/ | 7161 |
| 20 | clomifene citrate/ or *antiestrogen/ | 11245 |
| 21 | *diet therapy/ or *biological therapy/ | 19832 |
| 22 | *kinesiotherapy/ | 16269 |
| 23 | *diet/ | 77295 |
| 24 | exercise/ or *aerobic exercise/ or *exercise intensity/ or *high intensity exercise/ or *low intensity exercise/ or *moderate intensity exercise/ | 367595 |
| 25 | *yoga/ | 5116 |
| 26 | *cognitive therapy/ | 15216 |
| 27 | exp *psychotherapy/ or psychotherapy.mp. | 207153 |
| 28 | *behavior therapy/ or *cognitive behavioral therapy/ | 26371 |
| 29 | cbt.mp. | 23169 |
| 30 | *lifestyle/ or *lifestyle modification/ | 30825 |
| 31 | lifestyle intervention.mp. | 9631 |
| 32 | 8 or 9 or 10 or 11 or 12 or 13 or 14 or 15 or 16 or 17 or 18 or 19 or 20 or 21 or 22 or 23 or 24 or 25 or 26 or 27 or 28 or 29 or 30 or 31 | 1609349 |
| 33 | *body weight loss/ | 11372 |
| 34 | *anthropometric parameters/ or *body mass/ or *body weight/ or *fat mass/ or *waist circumference/ | 86313 |
| 35 | *body mass/ | 42651 |
| 36 | gestational diabetes/ or *maternal diabetes mellitus/ | 6785 |
| 37 | *luteinizing hormone/ | 24731 |
| 38 | *follitropin/ | 19382 |
| 39 | *high density lipoprotein cholesterol level/ or *low density lipoprotein cholesterol/ or *very low density lipoprotein cholesterol/ or *low density lipoprotein cholesterol level/ or *cholesterol level/ or *cholesterol/ or *total body cholesterol/ or *high density lipoprotein cholesterol/ or cholesterol.mp. or *total cholesterol level/ | 528632 |
| 40 | *androgen/ or free androgen index.mp. or *testosterone/ or *sex hormone binding globulin/ | 63680 |
| 41 | menstrual cycle/ or *menstrual rhythm/ or *menstruation/ or *ovary cycle/ | 49464 |
| 42 | ovulation/ | 28205 |
| 43 | exp *pregnancy rate/ | 5469 |
| 44 | spontaneous abortion/ or *abortion/ | 64866 |
| 45 | exp *live birth/ | 4010 |
| 46 | preterm birth.mp. or exp prematurity/ | 154082 |
| 47 | ovarian stimulation.mp. | 13828 |
| 48 | in vitro fertilization/ or *in vitro oocyte maturation/ or *oocyte retrieval/ or *ovulation induction/ | 30871 |
| 49 | fertilization/ | 38927 |
| 50 | glucose level.mp. or glucose blood level/ or exp *glucose level/ | 348014 |
| 51 | *hemoglobin A1c/ | 13925 |
| 52 | insulin level/ | 3544 |
| 53 | 33 or 34 or 35 or 36 or 37 or 38 or 39 or 40 or 41 or 42 or 43 or 44 or 45 or 46 or 47 or 48 or 49 or 50 or 51 or 52 | 1324726 |
| 54 | 7 and 32 and 53 | 5155 |
| 55 | limit 54 to yr="2022 -Current" | 675 |

Medline

| **#** | **Searches** | **Results** |
| --- | --- | --- |
| 1 | exp polycystic ovary syndrome/ | 18658 |
| 2 | ovarian degeneration.mp. | 49 |
| 3 | PCO.mp. | 5462 |
| 4 | sclerocystic ovarian degeneration.mp. | 2 |
| 5 | 1 or 2 or 3 or 4 | 23307 |
| 6 | *contraceptive agents, female/ or *contraceptives, oral/ or *contraceptive agents, hormonal/ | 19795 |
| 7 | Metformin/ | 18523 |
| 8 | exp androgen antagonists/ or *androgen receptor antagonists/ or *nonsteroidal anti-androgens/ or *estrogen antagonists/ | 23353 |
| 9 | exp *orlistat/ or exp *spironolactone/ | 4227 |
| 10 | *cyproterone/ or exp cyproterone acetate/ | 2296 |
| 11 | exp Flutamide/ or flutamide.mp. | 3904 |
| 12 | finasteride.mp. or exp Finasteride/ | 3699 |
| 13 | *Anti-Obesity Agents/ or sibutramine.mp. | 5480 |
| 14 | exp inositol/ or *inositol phosphates/ | 24593 |
| 15 | *contraceptive agents, female/ or exp contraceptives, oral/ or *contraceptives, oral, combined/ or *contraceptives, oral, hormonal/ or exp menstruation-inducing agents/ or exp contraceptive agents, hormonal/ | 62574 |
| 16 | exp Clomiphene/ | 5489 |
| 17 | fertility promoting agent.mp. | 1 |
| 18 | *Ovulation Induction/ or clomifene citrate.mp. | 7300 |
| 19 | exp *diet therapy/ or caloric restriction/ or diet, carbohydrate-restricted/ or diet, fat-restricted/ or diet, high-protein/ or diet, plant-based/ or diet, protein-restricted/ or diet, reducing/ | 47030 |
| 20 | *Exercise Therapy/ | 31666 |
| 21 | exercise/ or exp running/ or swimming/ or exp walking/ or exp warm-up exercise/ | 249954 |
| 22 | yoga.mp. or exp Yoga/ | 7696 |
| 23 | cognitive behavioral therapy/ or "acceptance and commitment therapy"/ or cognitive restructuring/ or mindfulness/ | 37920 |
| 24 | exp psychotherapy/ or exp behavior therapy/ or exp dance therapy/ or exp psychosocial intervention/ | 222600 |
| 25 | cbt.mp. | 15806 |
| 26 | exp life style/ or exp healthy lifestyle/ or exp life change events/ | 112882 |
| 27 | lifestyle intervention.mp. | 6450 |
| 28 | lifestyle modification.mp. | 5902 |
| 29 | 6 or 7 or 8 or 9 or 10 or 11 or 12 or 13 or 14 or 15 or 16 or 17 or 18 or 19 or 20 or 21 or 22 or 23 or 24 or 25 or 26 or 27 or 28 | 783869 |
| 30 | Weight Loss/ | 44951 |
| 31 | anthropometric parameter.mp. or exp Body Mass Index/ or *Anthropometry/ | 160099 |
| 32 | exp body size/ or *body weight/ or waist circumference/ | 389399 |
| 33 | *pregnancy complications/ or abortion, spontaneous/ or exp diabetes, gestational/ or *fetal macrosomia/ or fetal death/ or fetal diseases/ or hypertension, pregnancy-induced/ or maternal death/ or obesity, maternal/ | 164488 |
| 34 | exp follicle stimulating hormone/ or exp luteinizing hormone/ | 61777 |
| 35 | exp lipids/ or exp glycerides/ | 1303397 |
| 36 | Cholesterol, VLDL/ or *Cholesterol/ or Cholesterol, LDL/ or Cholesterol, HDL/ or cholesterol.mp. | 332741 |
| 37 | *Androgens/ or *Testosterone/ or *Sex Hormone-Binding Globulin/ or free androgen index.mp. or *Hyperandrogenism/ | 55253 |
| 38 | exp fertility/ or menstrual cycle/ or menstruation/ or exp pregnancy rate/ | 94254 |
| 39 | oocyte retrieval/ or ovulation induction/ | 14653 |
| 40 | exp live birth/ or exp stillbirth/ | 12048 |
| 41 | preterm birth.mp. or exp Premature Birth/ | 34797 |
| 42 | Ovarian stimulation.mp. | 7774 |
| 43 | Fertilization in Vitro/ | 37333 |
| 44 | exp Insulin/ or exp *Blood Glucose/ or glucose level.mp. | 250633 |
| 45 | 30 or 31 or 32 or 33 or 34 or 35 or 36 or 37 or 38 or 39 or 40 or 41 or 42 or 43 or 44 | 2410302 |
| 46 | 5 and 29 and 45 | 2850 |
| 47 | limit 46 to yr="2022 -Current" | 232 |

CNAHL

| **#** | **Query** | **Limiters/Expanders** | **Last Run Via** | **Results** |
| --- | --- | --- | --- | --- |
| S22 | S1 AND S12 AND S20 | Limiters - Publication Year: 2022-current Expanders - Apply equivalent subjects Search modes - Boolean/Phrase | Interface - EBSCOhost Research Databases Search Screen - Advanced Search Database - CINAHL Plus | Display |
|  |  |  |  |  |
| S21 | S1 AND S12 AND S20 | Expanders - Apply equivalent subjects Search modes - Boolean/Phrase | Interface - EBSCOhost Research Databases Search Screen - Advanced Search Database - CINAHL Plus | Display |
| S20 | S13 OR S14 OR S15 OR S16 OR S17 OR S18 OR S19 | Expanders - Apply equivalent subjects Search modes - Boolean/Phrase | Interface - EBSCOhost Research Databases Search Screen - Advanced Search Database - CINAHL Plus | Display |
| S19 | (MH "Insulins+") OR (MH "Insulin+") OR (MH "Blood Glucose") OR "insulin or blood glucose or ogtt or fasting insulin or" | Expanders - Apply equivalent subjects Search modes - Boolean/Phrase | Interface - EBSCOhost Research Databases Search Screen - Advanced Search Database - CINAHL Plus | Display |
| S18 | (MH "Ovulation+") OR (MH "Fertilization+") OR (MH "Fertility+") OR (MH "Oocyte Retrieval") OR (MH "Ovulation Induction") OR "( ovulation stimulation or fertility or ovulation or fertilization or oocyte yield or oocyte maturation or ) OR ( preterm birth or preterm delivery )" | Expanders - Apply equivalent subjects Search modes - Boolean/Phrase | Interface - EBSCOhost Research Databases Search Screen - Advanced Search Database - CINAHL Plus | Display |
| S17 | (MH "Androgens+") OR (MH "Hyperandrogenism") OR (MH "Testosterone+") OR "androgen or testosterone or shbg or free androgen index or hyperandrogenism" | Expanders - Apply equivalent subjects Search modes - Boolean/Phrase | Interface - EBSCOhost Research Databases Search Screen - Advanced Search Database - CINAHL Plus | Display |
| S16 | (MM "Lipoproteins, LDL Cholesterol") OR (MM "Lipoproteins, HDL Cholesterol") OR (MH "Lipids+") OR (MH "Cholesterol+") OR (MH "Lipoproteins, LDL+") OR (MH "Lipoproteins, HDL+") | Expanders - Apply equivalent subjects Search modes - Boolean/Phrase | Interface - EBSCOhost Research Databases Search Screen - Advanced Search Database - CINAHL Plus | Display |
| S15 | (MM "Follicle-Stimulating Hormone") OR (MM "Luteinizing Hormone") OR (MH "Gonadotropin-Releasing Hormone+") OR "follicle-stimulating hormone or FSH or luteinizing hormone or LH or gonadothropins" | Expanders - Apply equivalent subjects Search modes - Boolean/Phrase | Interface - EBSCOhost Research Databases Search Screen - Advanced Search Database - CINAHL Plus | Display |
| S14 | (MM "Diabetes Mellitus, Gestational") OR "( pregnancy or live birth or miscarriage or stillbirth or menstruation or menstrual regularity or abortion ) OR ( gestational diabetes or gdm or gestational diabetes mellitus or diabetes in pregnancy or pregnancy induced hypertention or maternal diabetes )" | Expanders - Apply equivalent subjects Search modes - Boolean/Phrase | Interface - EBSCOhost Research Databases Search Screen - Advanced Search Database - CINAHL Plus | Display |
| S13 | (MM "Body Mass Index") OR (MH "Body Weights and Measures+") OR (MH "Fat Free Mass") OR "weight loss or body weight or body fat or waist circumference or body mass index or BMI" | Expanders - Apply equivalent subjects Search modes - Boolean/Phrase | Interface - EBSCOhost Research Databases Search Screen - Advanced Search Database - CINAHL Plus | Display |
| S12 | S2 OR S3 OR S4 OR S5 OR S6 OR S7 OR S8 OR S9 OR S10 OR S11 | Expanders - Apply equivalent subjects Search modes - Boolean/Phrase | Interface - EBSCOhost Research Databases Search Screen - Advanced Search Database - CINAHL Plus | Display |
| S11 | (MM "Life Style Changes") OR (MH "Life Style+") OR "lifestyle intervention or lifestyle modification or lifestyle change" | Expanders - Apply equivalent subjects Search modes - Boolean/Phrase | Interface - EBSCOhost Research Databases Search Screen - Advanced Search Database - CINAHL Plus | Display |
| S10 | (MH "Psychotherapy+") | Expanders - Apply equivalent subjects Search modes - Boolean/Phrase | Interface - EBSCOhost Research Databases Search Screen - Advanced Search Database - CINAHL Plus | Display |
| S9 | (MH "Cognitive Therapy+") OR (MM "Cognitive Restructuring") OR (MH "Behavior Therapy+") OR (MH "Cognitive Therapy (Iowa NIC)+") OR (MH "Behavior Therapy (Iowa NIC)+") OR (MH "Cognitive Restructuring (Iowa NIC)") OR "cognitive behavioral therapy or cbt or behaviour therapy or cognitive restructuring" | Expanders - Apply equivalent subjects Search modes - Boolean/Phrase | Interface - EBSCOhost Research Databases Search Screen - Advanced Search Database - CINAHL Plus | Display |
| S8 | (MH "Warm-Up Exercise+") OR (MH "Therapeutic Exercise+") OR (MH "Aerobic Exercises+") OR "exercise therapy or exercise or running or jogging or walking or warm-up exercise or yoga" | Expanders - Apply equivalent subjects Search modes - Boolean/Phrase | Interface - EBSCOhost Research Databases Search Screen - Advanced Search Database - CINAHL Plus | Display |
| S7 | (MM "Diet, Fat-Restricted") OR (MH "Restricted Diet+") OR (MM "Diet, Sodium-Restricted") OR (MM "Diet, High Protein") OR (MM "Diet, Low Carbohydrate") OR (MH "Plant-Based Diet") OR (MH "Plant Proteins+") OR (MH "Diet Therapy+") | Expanders - Apply equivalent subjects Search modes - Boolean/Phrase | Interface - EBSCOhost Research Databases Search Screen - Advanced Search Database - CINAHL Plus | Display |
| S6 | (MH "Fertility Agents+") OR (MH "Clomiphene") OR "clomiphene or ovulation induction or clomifene citrate or fertility promoting agent" | Expanders - Apply equivalent subjects Search modes - Boolean/Phrase | Interface - EBSCOhost Research Databases Search Screen - Advanced Search Database - CINAHL Plus | Display |
| S5 | (MH "Inositol Phosphates+") OR (MH "Inositol+") | Expanders - Apply equivalent subjects Search modes - Boolean/Phrase | Interface - EBSCOhost Research Databases Search Screen - Advanced Search Database - CINAHL Plus | Display |
| S4 | (MH "Antiobesity Agents+") OR (MM "Sibutramine") OR (MM "Orlistat") OR (MM "Finasteride") OR "finasteride or antiobesity agent or orlistat or tetrahydrolipstatin or sibutramine" | Expanders - Apply equivalent subjects Search modes - Boolean/Phrase | Interface - EBSCOhost Research Databases Search Screen - Advanced Search Database - CINAHL Plus | Display |
| S3 | (MH "Androgen Antagonists+") OR "metformin or antiandrogen thearpy or sex hormone antagonist or spironolactone or cyproterone or cyproterone acetate or flutamide" | Expanders - Apply equivalent subjects Search modes - Boolean/Phrase | Interface - EBSCOhost Research Databases Search Screen - Advanced Search Database - CINAHL Plus | Display |
| S2 | (MH "Contraceptives, Oral+") OR (MM "Contraceptives, Oral Combined") OR (MH "Contraceptive Agents+") OR (MH "Menstruation Inducing Agents+") OR "( contraceptive agent or oral contraceptive pill or oral contraceptive agent ) OR menstrual inducing agent" | Expanders - Apply equivalent subjects Search modes - Boolean/Phrase | Interface - EBSCOhost Research Databases Search Screen - Advanced Search Database - CINAHL Plus | Display |
| S1 | "polycystic ovarian syndrome or pcos or polycystic ovary syndrome or stein leventhal syndrome or PCO or sclerocystic ovarian syndrome" OR (MM "Polycystic Ovary Syndrome") | Expanders - Apply equivalent subjects Search modes - Boolean/Phrase | Interface - EBSCOhost Research Databases Search Screen - Advanced Search Database - CINAHL Plus | Display |

Psychinfo

| **#** | **Searches** | **Results** |
| --- | --- | --- |
| 1 | polycystic ovary syndrome.mp. | 499 |
| 2 | polycystic ovarian syndrome.mp. | 118 |
| 3 | stein leventhal syndrome.mp. | 2 |
| 4 | pcos.mp. | 416 |
| 5 | PCO.mp. | 90 |
| 6 | 1 or 2 or 3 or 4 or 5 | 678 |
| 7 | exp Birth Control/ or exp Oral Contraceptives/ or contraceptive agent.mp. | 12300 |
| 8 | metformin.mp. | 670 |
| 9 | androgen antagonist.mp. or exp Antiandrogens/ | 241 |
| 10 | orlistat.mp. | 108 |
| 11 | spironolactone.mp. | 217 |
| 12 | exp Antiandrogens/ or cyproterone* cyproterone acetate.mp. | 235 |
| 13 | flutamide.mp. | 186 |
| 14 | finasteride.mp. | 203 |
| 15 | antiobesity agents.mp. | 10 |
| 16 | sibutramine.mp. | 177 |
| 17 | inositol.mp. | 1892 |
| 18 | inositol phosphate.mp. | 92 |
| 19 | clomiphene.mp. | 63 |
| 20 | fertility promoting agent.mp. | 0 |
| 21 | clomiphene citrate.mp. | 31 |
| 22 | ovulation induction.mp. | 49 |
| 23 | dietary treatment/ or exp nutrition/ or exp diets/ | 75650 |
| 24 | exercise therapy/ or exp physical therapy/ | 4306 |
| 25 | exp Exercise/ or exercise.mp. | 78443 |
| 26 | exp Yoga/ | 2544 |
| 27 | exp Behavior Therapy/ or exp Cognitive Behavior Therapy/ or exp Cognitive Therapy/ or cognitive behavio?r.mp. | 115923 |
| 28 | exp Psychotherapy/ | 219794 |
| 29 | cbt.mp. | 18309 |
| 30 | exp Lifestyle Changes/ or exp Lifestyle/ | 14376 |
| 31 | lifestyle intervention.mp. | 1310 |
| 32 | 7 or 8 or 9 or 10 or 11 or 12 or 13 or 14 or 15 or 16 or 17 or 18 or 19 or 20 or 21 or 22 or 23 or 24 or 25 or 26 or 27 or 28 or 29 or 30 or 31 | 489149 |
| 33 | exp Body Weight/ or exp Weight Loss/ or exp Weight Control/ or weigh loss.mp. | 62829 |
| 34 | exp Anthropometry/ or exp Body Mass Index/ or anthropometric parameter.mp. | 9224 |
| 35 | exp Body Fat/ or exp Body Mass Index/ or exp Body Weight/ or waist circumference.mp. or exp Insulin/ | 72504 |
| 36 | gestational diabetes/ | 332 |
| 37 | obstetrical complications/ or exp birth/ or exp caesarean birth/ or exp pregnancy/ or exp premature birth/ | 50304 |
| 38 | spontaneous abortion/ or exp pregnancy outcomes/ | 24527 |
| 39 | exp Birth Rate/ or exp Fertility/ or live birth.mp. | 4254 |
| 40 | menstrual cycle/ or exp menstruation/ or exp ovulation/ | 4654 |
| 41 | exp Luteinizing Hormone/ or exp Follicle Stimulating Hormone/ | 877 |
| 42 | cholesterol.mp. or exp Cholesterol/ | 9916 |
| 43 | lipids/ or exp lipoproteins/ | 4720 |
| 44 | exp Androgens/ or exp Testosterone/ or free androgen index.mp. | 7962 |
| 45 | exp ovulation/ or exp menstrual cycle/ | 4654 |
| 46 | oocyte yield.mp. | 2 |
| 47 | stillbirth.mp. | 837 |
| 48 | ovarian stimulation.mp. | 34 |
| 49 | insulin.mp. or exp Insulin/ | 12567 |
| 50 | blood glucose.mp. or exp Blood Sugar/ | 7765 |
| 51 | 33 or 34 or 35 or 36 or 37 or 38 or 39 or 40 or 41 or 42 or 43 or 44 or 45 or 46 or 47 or 48 or 49 or 50 | 154429 |
| 52 | 6 and 32 and 51 | 68 |
| 53 | limit 52 to yr="2022 -Current" | 7 |
