# Supplementary material for: The effect of lifestyle interventions in women with polyendocrine metabolic ovarian syndrome: A systematic review and meta-analysis
Source: PLoS Med. 2026 Sep 23;23(9):e1004887. doi: 10.1371/journal.pmed.1004887 (PMC13600514; doi:10.1371/journal.pmed.1004887)
Supplement: Supplementary file 3 [file pmed.1004887.s003.docx]

S2 Appendix: Characteristics of included studies

| **Author, year,**  **country** | **Population/**  **Setting** | **Age**  **(mean ± SD)** | **Study**  **Design** | **Sample Size per**  **group** | **Intervention/ exposure**  **details** | **Comparison/**  **control**  **details** | **Follow up**  **Duration** | **Outcomes** |
| --- | --- | --- | --- | --- | --- | --- | --- | --- |
| Colonetti et al., 2025 | 29 Women over 18 years old diagnosed with PCOS | 27.17 ± 5.28 | Randomised controlled trial | 14 in the intervention are  15 in the control arm | Low carbohydrate diet + strength exercise | Strength exercise alone | 12 weeks | Total testosterone |
| Meenakshi et al, 2024, India | 30 females with PCOS selected from Private Institute | 25.5 ± 4.75 | Randomised controlled trial | 15 | resistance training group received exercises with dumbbells | Swiss ball training group received exercises with Swiss ball, in addition to aerobic exercise | 8 weeks | BMI (Body Mass Index), Abdominal girth, and Menstrual Irregularity Questionnaire (MIQ)^12^ were used as outcome measures. It measures obesity, waist circumference and menstrual activity respectively. |
| Deshmukh et al, 2023, UK | 40 women from Academic Diabetes, Endocrinology and Metabolism research centre at Hull Royal Infirmary. | 27.9 ± 4.7 | Randomised controlled trial | VLCD (*n* = 11) or a conventional energy deficit approach (*n* = 11) | For the first eight weeks, the VLCD group was instructed to follow a prescription of 800 kcal a day (irrespective of their baseline body weight), in the form of soups and drinks made from pre-prepared sachets provided by the Cambridge Weight PlanTM company, and support and ifromation regarding the consumption of food replacement sachets | energy deficit approach group acted as the control group in this trial. The kcal prescription was bespoke for each patient and calculated using the Henry equation based on gender, age, and weight to ascertain basal metabolic rate [23], which was then multiplied by physical activity level  **Both groups received** dietetic support and education | 8 weeks | Body weight, waist circumference, SHBG, fasting blood glucose, HOMA-IR, FAI, weight |
| Nasiri et al, 2022, Iran | 45 women with PCOS divided into three groups receiving HIIT (n=15), COM interventions (n=15) or control group (n=15)  patients referring to the gynecological clinic of Imam Reza  Hospital, Kermanshah, Iran | 24.13 ± 5.4 | Randomised contolled trial | 15 | HIIT program included intermittent running with 100% MAV and 50% MAV.  COM training group, the resistance training program consisted 24 sessions of selected resistance exercises during eight weeks holding 3 sessions each week | control group were also enquired to do only their normal daily routines and avoid doing any physical activities throughout the training progra | 8 weeks | BMI, weight, WHR, |
| **Meneghini et al, 2023, Italy** | 84 patients seeking infertility treatment at the Complex Organizational Unit (UOC) of Reproductive Pathophysiology and Andrology, which carries out its activities within the Sandro Pertini Hospital (Rome, Italy), were recruited | 33.88 ± 4.0 | Randomised controlled trial | 42 patients received VLCKD therapy treatment and 42 patients received Mediterranean diet therapy treatment | VLCKD – intensive phase protocol for 60 days; substitute food e.g. protein and carbohydrate sources already balanced specifically for this regimen – plant based and animal proteins  First 3 days – addition of MCT oil | Low-calorie Mediterranean regimen including 1400kcal for 90 days  Rice and pasta allowed twice a week | 90/120 days depending on outcome | anthropometric measures (body mass index (BMI), waist and hip circumferences, waist-to-hip ratio (WHR), and abdominal circumference); hormone levels (luteinizing hormone (LH), follicle-stimulating hormone (FSH), FSH/LH ratio, estradiol, , androstenedione, testos- terone); metabolic parameters (cholesterol, high-density lipoprotein (HDL), triglycerides); and reproductive parameters (menstrual regularity, antral follicle count (AFC), AMH, and risk of developing OHSS). |
| Furtardo et al, 2024, Brazil | 56 women with PCOS from our previously reported clinical trials involving resistance [10,26] and aerobic exercise | 28.9 ± 5.1 | Randomised controlled trial | 30 women with PCOS were included in the resistance training and 26 in the continuous aerobic training | Briefly, the resistance and continuous aerobic training were performed three times a week, in 50-minute to one-hour sessions for 16 wks, under supervision by physical education professionals. For the resis- tance exercise, a linear periodization of training protocol was used, with decreasing volume and increasing intensity according to [32,33] and the resistance exercises included bench press, leg extension, front lat pull-down, leg curl, lateral raise, leg press (45°), triceps pulley, calf leg press, arm curl, and abdominal exercise. | The aerobic exercise was carried out on a treadmill (Embreex 570-L and Embreex 570-Pro, SC, Brazil) with a progressive increase in the dura- tion of the training, from 30 minutes in the first week to 50 minutes in the last | 16 weeks | **BMI (kg/m2** **WC (cm)**  **WHR** **Testosterone (ng/dL)** **FAI**  SHBG  FSH  LH  HDL  LDL  Triglycerides  Fasting insulin  HOMA-IR  Fasting cholesterol |
| Bozbulut et al, 2024, Turkey | 40 adoles- cents diagnosed with PCOS between the ages of 13–18 and volunteered to participate | 15.85 ± 1.58 | Randomised controlled trial | 20  randomly assigned to two different diet groups, control or experimental diet (the control and the RESMENA groups, respectively), by the research assistant with an allocation ratio of 1:1, during the study period | characterized by a higher meal frequency, consisting of seven meals/d (including breakfast, lunch, dinner, and two snacks in the morning and two snacks in the afternoon), and by a different macronutrient distribution – 40% total energy value from carbohydrates, 30% from proteins, and 30% from lipids | It was based on the American Heart Association (AHA) guide- lines [20], including 3–5 meals per day, macronutrient distri- bution of 55% total energy value from carbohydrates, 15% proteins, and 30% lipids, a healthy FA profile, and a cholesterol consumption lower than 300 mg/day. | 6 months | The primary outcomes were changes in androgen profile and IR. Secondary outcomes included changes in anthropometric measurements, body composition, lipid profile, and inflammatory profile. |
| Tripathi et al., 2023, India | 50 patients who visited the author's clinic with Polycystic Ovary Syndrome or Disease from November 2022 to April 2023 | 26.23 ± 5.25 | Randomised controlled trial | 25 in wolly group, 25 in control group | women-centric fitness program with dance movements, strengthening core and pelvic muscles. | control group did not receive the Wolly workout program | 3 months | Waist, weight, BMI, testosterone, cholesterol, triglycerides, HDL, LDL, fasting glucose |
| Dietz de Loos et al., 2023, Netherlands | 183 women aged between 18-38 years, with (BMI) > 25 kg/m^2^, attending the division of Reproductive Endocrinology and Infertil ity of the Department of Obstetrics and Gynaecology, at the Erasmus MC, the Netherlands | Median 29 (IQR 26–32) | Randomised controlled trial | SMS+ group (n=60)  SMS- group (n=63)  CAU group (n=60) | twenty 2.5 h group meetings over the period of one-year covering normo-caloric diet, as recommended by the “Dutch Food Guide”  exercise according to the “Global Recommendations for physical activity by the World Health Organization”,  and cognitive behavioral therapy  +/- SMS | Care as usual | 1 year | Conception, hypertensive disorders in pregnancy, GDM, live birth |
| Philbois et al., 2022, Brazil | 126 participants aged between 18 and 39 years were enrolled, screened at the Gynecology and Obstetrics Clinic of the Ribeirão Preto Medical School’s Hospital (HCFMRP/USP) | 29 ± 4.7 | Randomised controlled trial | Control, MICT, HIIT (25 in each) | 16 weeks of aerobic physical training | control group did not undergo exercise, | 16 weeks | HOMA-IR, insulin, cholesterol, triglyceride, glucose, HDL, LDL, testosterone, BMI |
| **Niranjani et al,** 2022, India | Girls from different colleges in chennai screened for PCOs and underwent OGTT  50 PCOS girls who had impaired glucose level and who are ready to give consent for the procedure and the interventions were selected for the study. self-administered questionnaire was used to elicit the baseline data | 24.23 ± 3.69 | Randomised controlled trial | 50 in each group – control group (no intervention), experimental group 1 (all three interventions), experimental group 2 (selected interventions) | Experimental group 1 (n=50): cinnamon tea, exercise programme, counselling  Experimental group 2 (n=50): exercise programme, counselling | Control group(n=50):only education related to PCOS | 6 months | Fasting blood sugar |
| Patten et al, 2022, Australia | Recuitment through community and social media advertising | 29.7 ± 4.8 | Rndomsied controlled trial | 15 (HIIT) 14(MICT) | 3 sessions per week for 12 weeks | 3 sessions per week for 12 weeks | 12 weeks | Menstrual regularity, testosterone, SHBG, glucose, insulin, waist circumference, weight, BMI |
| Mei et al, 2022, China | 72 participants with PCOS who were attending the Department of Traditional Chinese Medicine | 28.02 ± 6.21 | Randomised controlled trial | 36 in each group | Mediterranean diet – combination of Mediterranean and low-card diet; max carb intake = 100g and increased protein and fat in the day; whole grain staple food, advised to have fruits, vegetables, cereals, extra virgin olive oil | Low-fat diet – no fatty foods e.g. fatty meats, fried foods, offal etc  Increased intake of cereals, vegetables, fruits | 12 weeks | Body weight, BMI, waist circumference, waist-hip ratio, insulin, cholesterol, fasting glucose, HDL, LDL, total testosterone, FSH. LH, triglycerides |
| D’souza et al, 2022, India | women diagnosed with PCOS were selected through a purposive sampling technique, from two tertiary settings | 24.26±3.84 | Randomised controlled trial | 15 in each group | counselled for an insulin resistance diet  diet menu was prepared with the help of a dietician based on the BMI.  Brisk walk for 30 minutes first one month followed by jogging from the month onwards for the same duration was advised.  Core muscle exercises, half push- ups, and four types of burpees (beginners, basic, squat thrust, and mountain climber) were demonstrated  Complaince chart, videos, reminders, warm up cool down | continued the regular medical treatment | 6 months | BMI, hirsutism, fasting blood sugar, wait hip ratio, menstrual pattern |
| Nikrad et al, 2023, | 48 women with obesity and affected by PCOS from the infertility clinic of Al-Zahra hospital and Sheykholrais Polyclinic in Tabriz, Iran were recruited based on rotterdam criteria diagnosis by gynaecologist | 31.95 ± 2.59 | Randomised controlled trial | thylakoid (N = 21) placebo groups (N = 23) | 5 g/day thylakoid‐rich spinach extract  personalized hypocaloric diet with 500 calories less than the total energy expenditure was prescribed to all patients. | placebo (5 g cornstarch) along with prescribed low‐calorie diet.  personalized hypocaloric diet with 500 calories less than the total energy expenditure was prescribed to all patients. | 12 weeks | LPS, neurotrophic factors such as BDNF and S100B levels  FBG, insulin and HOMA-IR and hormonal profiles, including the FSH/LH ratio, FTI and DHEA-S |
| Cincione et al, 2022, Italy | subjects were enrolled at the outpa- tient Endocrinology clinic, and included 144 treatment-naïve women that were affected by PCOS attending the Outpa- tient Clinic of the Unit of Endocrinology in our Department. | 33.5 ± 5.3 | Randomised controlled trial | 73 in the KD group  71 in the MD group | Mixeed ketogenic diet – daily protein intake, and complete amino acid com- position profile with near-zero carbohydrates and fat con- tent, | D diet was rich in whole grains (pasta, bread, and whole wheat), eggs, poultry, fish, vegetables, legumes, fruits and olive oil as the main condiment, and low in red and processed meat | 45 days | Body weight, BMI, WC, WHR, glucose, insulin, HOMA-IR, LH, FSH, LH/FSH, free testosterone, total tesosterone, SHBG |
| Almenning et al. 2015 Norway | Women with PCOS; Medical Center | 27.2 ± 5.5 | Parallel RCT | Intervention (high intensity interval training = 10 and strength training =11) Control = 10 | 3 weekly exercise sessions for 10 weeks with normal diet (high intensity interval training did high interval training, strength training did strength training) | Maintain normal diet and physical activity | 10 weeks | testosterone, SHBG, FAI, total cholesterol, HDL-C, LDL-C, triglycerides, glucose, insulin, weight, BMI, waist circumference |
| Brown et al. 2009 USA | Women with PCOS aged 18 to 50; Medical Cente | Median ~33 | Parallel RCT | Intervention = 21 Control = 16 | 8-12 week ramp up followed by 12-week moderate intensity exercise | no change in lifestyle | 12 weeks for control, 20-24 weeks for intervention | testosterone, lipid profile, fasting glucose and insulin, weight, BMI, waist/hip ratio |
| Costa et al. 2018, Brazil | Women with PCOS; Medical Center | 26.0 ± 4.75 | Parallel RCT | Intervention =14 Control=13 | 16-weeklong supervised aerobic training three times per week | No exercise intervention n | 16 weeks | Health-related quality of life, cardiorespiratory fitness, cardiometabolic profile, and affective response |
| Guzick et al. 1994 USA | Women with PCOS aged 20 to 40 years and Obese; Medical Center | 32 | Parallel RCT | Intervention = 6 Control = 6 | 12-week behavioral weight control program | No treatment | 12 weeks | Total testosterone, SHBG, non-SHBG testosterone, height, weight, body fat distribution, fasting glucose, insulin |
| Hoeger et al. 2004 USA | Women with PCOS and BMI > 25 kg/m2; Research Center | 28.5 ± 5.3 | Parallel RCT | Intervention = 11 Control = 9 | Lifestyle intervention with placebo (individualized meal plan and exercise plan) | No lifestyle interventio n | 48 weeks | menstrual diaries, testosterone, SHBG, FAI, F-G score, height, weight, BMI, waist/hip circumference, OGTT glucose, lipid profile, OGTT insulin |
| Hoeger et al. 2008 USA | Women with PCOS ages 12 to 18 and obese; Research Center | 15.9 ± 1.9 | Parallel RCT | Intervention: 11 Control = 11 | Lifestyle intervention (training classes on diet, exercise, and behavior modification skills) | Standard office advice on nutrition and exercise | 24 weeks | menstrual cycle average per 24 weeks, total and free testosterone, SHBG, FAI, hirsutism, OGTT glucose, lipid profile, OGTT insulin, BMI, waist circumference |
| Stener Victorian et al, 2013 Sweden | Women with PCOS age 18 to 30; Academic Medical Center | 29.9 ± 4.4 | Parallel RCT | Intervention = 34 Control = 17 | Weight maintenance, moderate exercise | No exercise | 16 weeks | Menstrual pattern, total testosterone, SHBG, free testosterone, FAI, F-G score, lipid profile, fasting glucose and insulin, height, weight, BMI, WHR |
| Kiel et al. 2021 Norway and Australia | Women with PCOS; Academic Medical Center | 29.6 ± 5.1 | Parallel RCT | Interventions (low intensity interval training [LV]-; high intensity interval training [HIT] = 21 HV-HIT = 20) Control = 23 | 16-week low-volume HIT workout, or 16-week high volume HIT workout followed by 36-week home-based HIT | Nonexerci sing | 12 months | Menstrual frequency (primary), markers of cardiometabolic and reproductive health, quality of life, adherence to and enjoyment of HIT |
| Kogurea et al. 2020 Brazil | Women with PCOS; Academic Medical Center | 28.9 ± 5.1 | Parallel RCT | Interventions (continuous aerobic training = 37 intermittent aerobic training = 35) Control = 38 | 16 weeks of continuous aerobic training or 16 weeks of intermittent aerobic training | Non training | 16 weeks | testosterone, FAI, SHBG, height, weight, hip circumference, waist circumference, WHR, actual BMI |
| Moeller et al., 2018 Denmark | Women with PCOS; Academic Medical Center | Median ~30.5 | Parallel RCT | Intervention (motivational interviews) = 19 Control = 18 | Motivational interviews were conducted once every 2 weeks (12 total times) | Standard care (no extra appointme nts, patients seen initially and at follow up only) | 6 months | Weight, hip and waist circumference, WHO-5, MDI, Sf-36, PCOS-Q, BMI |
| Nasrekani et al. 2016 Iran | Women with PCOS; Medical Center | 24.13 ± 5.4 | Parallel RCT | Intervention = 10 Control = 10 | 3 days/week of aerobic training | No intervention n | 12 weeks | Weight, BMI |
| Oberg et al., 2018, Sweden | Women with PCOS who were overweight/ob ese (BMI ≥ 27 kg/m2)/ Teaching hospital | 30.45 ± 5.4 | Parallel RCT | Intervention = 34 Control = 34 | Group meetings 3 per month where they were counseled on weight control, personal leadership, mindfulness, physical activity and diet. Meetings incorporated goal setting, stimulus control, problem‐ solving and stress management techniques to aid in behavioral change. One-on-one coaching sessions 1 per month to discuss individual training regimens and diet changes | General healthy lifestyle rec‐ commend ations given by a midwife and supported by a pamphlet with written advice about diet and exercise | Intervention and control groups occurred in parallel for 4 months; however, after 4 months, the control group undertook the intervention (again for 4 months) and the entire study sample was followed up at 12 months | Weight, BMI, fat mass, lean body mass, testosterone, SHBG, FAI, menstrual function |
| Ribeiro, 2019 and 2021 and 2021, Brazil (2 publications reporting on the same study) | Women with PCOS; Academic Medical Center | 28.87 ± 5.13 | Parallel RCT | Interventions (Continuous aerobic training = 28 Intermittent aerobic training = 29) Control = 15 | Both continuous and intermittent aerobic training on 3 days per week for 40-60 minutes with a 5-minute warm-up and 5 minutes cool down between 50%- 60% of the maximum heart rate For specific protocols of continuous and intermittent training sessions (which varied each week) see Table 1 of the published manuscript | No training | 4 months | WC, HC, WHR, fat mass, lean body mass, weight, BMI, testosterone, SHBG, fasting insulin, fasting glucose, HDL-C, TC, TG, LDL-C, FAI |
| Saremi et al., 2013 Iran | Women with PCOS; Medical Center | 24.35 ± 4.41 | Parallel RCT | Intervention = 11 Control = 11 | Aerobic training 3 days/week for 40-60 min each | Asked not to do more physical activity then they used to | 8 weeks | Total testosterone, HDL-C, LDL-C, triglycerides, fasting glucose, fasting insulin, weight, BMI, waist circumference, WHR |
| Stefanaki et al., 2015 Greece | Women with PCOS aged 15 to 40; Academic Medical Center | Intervention 23.4 ± 4.62; control 28.3 ± 7.2 | Parallel RCT | Intervention = 23 Control = 23 | 8-week mindfulness stress management program | No interventio n | 8 weeks | BMI, |
| Turan et al. 2015 Turkey | Women with PCOS and BMI < 25 kg/m2; Academic Medical Center | 24.45 ± 2.8 | Parallel RCT | Intervention = 16 Control = 16 | Structured exercise programs 3 times/week (aerobic and resistance exercise) | General dietary and behavioral advice | 8 weeks | Menstrual cycle, total testosterone, free testosterone, total cholesterol, HDL-C, LDL-C, triglycerides, fasting glucose, fasting insulin, BMI, waist circumference |
| Vigorito et el. 2007 Italy | Women with PCOS and overweight; Academic Medical Center | 21.7 ± 2.3 | Randomised control trial | Intervention = 45 Control = 45 | Structured supervised training sessions 3 times/week | No training program | 12 weeks | Menses diary, testosterone, SHBG, FAI, height, weight, BMI, waist circumference, WHR, lipid profile, fasting glucose and insulin, |
| Kabiri et al., 2024 | Overweight or obese (BMI ≥25 kg/m²) women of reproductive age (18–45 years) with polycystic ovary syndrome | MIND diet group: 30.85 ± 5.33 years  Control group: 28.92 ± 4.33 years | Randomised controlled trial | Intervention group = 26  Control group = 26 | Low-calorie MIND diet (modified energy deficit of 500–750 kcal/day) | Low-energy diet | 8 weeks | Weight, BMI, waist circumference |
