# Supplementary material for: The effect of lifestyle interventions in women with polyendocrine metabolic ovarian syndrome: A systematic review and meta-analysis
Source: PLoS Med. 2026 Sep 23;23(9):e1004887. doi: 10.1371/journal.pmed.1004887 (PMC13600514; doi:10.1371/journal.pmed.1004887)
Supplement: Supplementary file 4 [file pmed.1004887.s004.docx]

S3 Appendix: Risk of bias assessment

| Study | Randomisation process | Effect of assignment to intervention | Effect of adhering to intervention | Missing outcome data | Measurement of the outcome | Selection of the reported data | Overall risk |
| --- | --- | --- | --- | --- | --- | --- | --- |
| Meenakshi et al., 2024 | Low | Low | Low | Low | Low | Low | Low |
| Deshmukh et al., 2023 | Low | Some concerns | High | Some concerns | Low | Low | High |
| Nasiri et al, 2022 | Low | Some concerns | Low | Low | Low | Low | Some |
| Meneghini et al., 2023 | High | Some concerns | Some concerns | Low | Low | Low | High |
| Furtardo et al, 2024 | Low | Low | Low | Low | Low | Low | Low |
| Bozbulut et al, 2024 | Low | Low | High | Some concerns | Low | Low | High |
| Tripathi et al., 2023 | High | Low | Low | Low | Low | Low | High |
| Dietz de Loos et al., 2023 | Low | Low | High | Some concerns | Low | Low | High |
| Philbois et al., 2022 | Low | High | Some concerns | Some concerns | Low | Low | High |
| Niranjani et al, 2022 | High | Low | Some concerns | Low | Low | Low | High |
| Patten et al, 2022 | Low | Low | Low | Low | Low | Low | Low |
| D’souza et al, 2022 | High | Low | Low | Low | Low | Low | High |
| Nikrad et al, 2023 | Low | Low | Low | Low | Low | Low | Low |
| Cincione et al, 2022 | Low | Low | Some concerns | Low | Low | Low | Some concern |
| Mei et al, 2022 | Low | Low | Low | Low | Low | Low | Low |
| Almenning et al., 2015 | Low | High | Low | Low | Low | Low | Some concern |
| Brown et al., 2009 | Low | Low | Low | High | Low | Some concerns | High |
| Costa et al., 2018 | High | High | Some concerns | Low | Low | Low | High |
| Guzick et al., 1994 | Low | High | Some concerns | Low | Low | Low | High |
| Hoeger et al., 2004 | Low | Low | Low | Some concerns | Low | Low | Some concern |
| Hoeger et al., 2008 | Low | Low | Low | Low | Low | Low | Low |
| Kiel et al., 2021 | Low | Some concerns | Some concerns | Low | Low | Low | Some concern |
| Kogurea et al., 2020 | Low | High | Some concerns | Low | High | Low | High |
| Moeller et al., 2018 | Low | Low | Low | Some concerns | High | Low | Some concern |
| Nasrekani et al.. 2016 | Some concerns | Some concerns | Some concerns | Low | Some concerns | Some concerns | Some concern |
| Oberg et al., 2018 | Low | Low | Low | Low | Low | Low | Low |
| Ribeiro, 2019 and 2021 (2 publications reporting on the same study) | Low | Some concerns | Low | Low | Low | Low | Some concern |
| Saremi et al., 2013 | Some concerns | Some concerns | Some concerns | Low | Some concerns | Some concerns | Some concern |
| Stefanaki et al., 2015 | Low | Low | Low | Low | Low | Low | Low |
| Stener Victorian 2013 | Low | Some concerns | Some concerns | Low | Low | Low | Some concern |
| Turan et al., 2015 | Low | Some concerns | Low | Low | Low | Low | Some concern |
| Vigorito et el., 2007 | Low | Low | Low | Low | Low | Low | Low |
| Colonetti et al., 2025 | Low | Low | Low | Low | Low | Low | low |
| Kabiri et al., 2024 | Low | Low | Low | Low | Low | Low | Low |
