# Supplementary material for: The effect of lifestyle interventions in women with polyendocrine metabolic ovarian syndrome: A systematic review and meta-analysis
Source: PLoS Med. 2026 Sep 23;23(9):e1004887. doi: 10.1371/journal.pmed.1004887 (PMC13600514; doi:10.1371/journal.pmed.1004887)
Supplement: Supplementary file 5 [file pmed.1004887.s005.docx]

S4 Appendix: GRADE assessment of lifestyle vs minimal treatment

|  | **Quality assessment** | | | | | | **No. Participants** | |  | | | |
| --- | --- | --- | --- | --- | --- | --- | --- | --- | --- | --- | --- | --- |
| **No studies** | **Design** | **Risk of bias** | **Inconsistency** | **Indirectness** | **Impression** | **Other** | **Intervention** | **Control** | **Effect, random**  **[95% CI]** | **Favours** | **Certainty** | **Importance** |
| Outcome: Weight | | | | | | | | | | | | |
| 21 | RCT | Serious | No serious inconsistency | Serious indirectness | Serious imprecision | None |  |  |  | No difference | ⨁⨁◯◯  LOW | Critical |
| Outcome: BMI | | | | | | | | | | | | |
| 28 | RCT | Serious | No serious inconsistency | Serious indirectness | No serious imprecision | None |  |  |  | No difference | ⨁⨁⨁◯  MODERATE | Critical |
| Outcome: Waist circumference | | | | | | | | | | | | |
| 18 | RCT | Serious | No serious inconsistency | Serious indirection | No serious imprecision | None |  |  |  | Lifestyle treatment | ⨁⨁⨁◯  MODERATE | Critical |
| Outcome: Waist-hip ratio | | | | | | | | | | | | |
| 14 | RCT | Serious | No serious inconsistency | Serious indirections | Serious imprecision | None |  |  |  | No difference | ⨁⨁◯◯  LOW | Critical |
| Outcome: SHBG | | | | | | | | | | | | |
| 14 | RCT | Serious | No serious inconsistency | Serious indirections | Serious imprecision | None |  |  |  | No difference | ⨁⨁◯◯  LOW | Critical |
| Outcome: Total testosterone | | | | | | | | | | | | |
| 19 | RCT | Serious | Serious inconsistency | Serious indirections | Serious imprecision | None |  |  |  | No difference | ⨁◯◯◯  Very LOW | Critical |
| Outcome: Free Androgen Index | | | | | | | | | | | | |
| 12 | RCT | Serious | Serious inconsistency | Serious indirections | No serious imprecision | None |  |  |  | No difference | ⨁◯◯◯  Very LOW | Critical |
| Outcome: Fasting glucose | | | | | | | | | | | | |
| 22 | RCT | Serious | No serious inconsistency | No serious indirections | serious imprecision | None |  |  |  | Lifestyle treatment | ⨁⨁⨁◯  MODERATE | Critical |
| Outcome: Fasting insulin | | | | | | | | | | | | |
| 18 | RCT | Serious | No serious inconsistency | No serious indirections | serious imprecision | None |  |  |  | Lifestyle treatment | ⨁⨁⨁◯  MODERATE | Critical |
| Outcome: Total cholesterol | | | | | | | | | | | | |
| 17 | RCT | Serious | No serious inconsistency | Serious indirections | Serious imprecision | None |  |  |  | No difference | ⨁⨁◯◯ LOW | Critical |
| Outcome: HDL-C | | | | | | | | | | | | |
| 15 | RCT | Serious | No serious inconsistency | Serious indirections | No serious imprecision | None |  |  |  | No difference | ⨁⨁⨁◯  MODERATE | Critical |
| Outcome: LDL-C | | | | | | | | | | | | |
| 15 | RCT | Serious | No serious inconsistency | Serious indirections | No serious imprecision | None |  |  |  | No difference | ⨁⨁⨁◯  MODERATE | Critical |
| Outcome: TG | | | | | | | | | | | | |
| 16 | RCT | Serious | No serious inconsistency | Serious indirections | No serious imprecision | None |  |  |  | No difference | ⨁⨁⨁◯  MODERATE | Critical |
