# Supplementary material for: The effect of lifestyle interventions in women with polyendocrine metabolic ovarian syndrome: A systematic review and meta-analysis
Source: PLoS Med. 2026 Sep 23;23(9):e1004887. doi: 10.1371/journal.pmed.1004887 (PMC13600514; doi:10.1371/journal.pmed.1004887)
Supplement: Supplementary file 6 [file pmed.1004887.s006.docx]

S5 Appendix- Subgroup analysis

Appendix 5a: Subgroup analysis based on type of lifestyle intervention

Effect of different types of lifestyle interventions on waist-hip ratio

Effect of different types of lifestyle interventions on sex-hormone binding globulin

Effect of different types of lifestyle interventions on total testosterone

Effect of different types of lifestyle interventions on free androgen index

Effect of different types of lifestyle interventions on total cholesterol

Effect of different types of lifestyle interventions on high-density lipoproteins

Effect of different types of lifestyle interventions on low-density lipoproteins

Effect of different types of lifestyle interventions on triglyceride.

Appendix 5b: Subgroup analysis based on duration of lifestyle intervention

Effect of different durations of lifestyle interventions on weight.

Effect of different durations of lifestyle interventions on BMI.

Effect of different durations of lifestyle interventions on waist-hip ratio.

Effect of different durations of lifestyle interventions on SHBG.

Effect of different durations of lifestyle interventions on total testosterone.

Effect of different durations of lifestyle interventions on FAI.

Effect of different durations of lifestyle interventions on total cholesterol.

Effect of different durations of lifestyle interventions on high-density lipoproteins.

Effect of different durations of lifestyle interventions on low-density lipoproteins.

Effect of different durations of lifestyle interventions on triglycerides.

Appendix 5c: Subgroup analysis based on baseline weight categories of participants

Effect of lifestyle intervention on BMI stratified by baseline weight.

Effect of lifestyle intervention on waist circumference stratified by baseline weight.

Effect of lifestyle intervention on SHBG stratified by baseline weight.

Effect of lifestyle intervention on FAI stratified by baseline weight.

Effect of lifestyle intervention on high-density lipoproteins stratified by baseline weight.

Effect of lifestyle intervention on triglycerides stratified by baseline weight.
